# Mechanism of microbial production of acetoin and 2,3-butanediol optical isomers and substrate specificity of butanediol dehydrogenase

**DOI:** 10.1186/s12934-023-02163-6

**Published:** 2023-08-29

**Authors:** Yuchen Li, Xiangying Zhao, Mingjing Yao, Wenli Yang, Yanlei Han, Liping Liu, Jiaxiang Zhang, Jianjun Liu

**Affiliations:** 1 Shandong Food Ferment Industry Research & Design Institute, Qilu University of Technology, Shandong Academy of Sciences), Jinan, 250013 China; 2 School of Food Science and Engineering, Qilu University of Technology (Shandong Academy of Sciences), Jinan, 250353 China

**Keywords:** Acetoin, 2,3-butanediol, Butanediol dehydrogenases, Optical isomer, Optical purity

## Abstract

3-Hydroxybutanone (Acetoin, AC) and 2,3-butanediol (BD) are two essential four-carbon platform compounds with numerous pharmaceutical and chemical synthesis applications. AC and BD have two and three stereoisomers, respectively, while the application of the single isomer product in chemical synthesis is superior. AC and BD are glucose overflow metabolites produced by biological fermentation from a variety of microorganisms. However, the AC or BD produced by microorganisms using glucose is typically a mixture of various stereoisomers. This was discovered to be due to the simultaneous presence of multiple butanediol dehydrogenases (BDHs) in microorganisms, and AC and BD can be interconverted under BDH catalysis. In this paper, beginning with the synthesis pathways of microbial AC and BD, we review in detail the studies on the formation mechanisms of different stereoisomers of AC and BD, summarize the properties of different types of BDH that have been tabulated, and analyze the structural characteristics and affinities of different types of BDH by comparing them using literature and biological database data. Using microorganisms, recent research on the production of optically pure AC or BD was also reviewed. Limiting factors and possible solutions for chiral AC and BD production are discussed.

**Supplementary Information:**

The online version contains supplementary material available at 10.1186/s12934-023-02163-6.

## Introduction

In the 21st century, limited resources, lack of energy, and pollution of the environment are making it harder to develop in a sustainable way. This is forcing traditional industrial manufacturing fields such as the chemical, food, and pharmaceutical industries to devolp new ways to make their products using renewable energy. Using microorganisms or enzymes with biocatalytic activities in manufacturing offers novel approaches for this innovation and is slowly making its way into more conventional sectors [1, 2]. In particular, the biomanufacturing process can operate in a mild reaction environment utilizing renewable resources and improve enantiomer selectivity through manipulation of the biosynthetic pathway. The advanced production method exemplified by biomanufacturing is a model for industrial development that solves the problem of resource depletion and environmental pollution at its source. It plays a crucial role in the sustainable development of industrial production and is a topic of intense research interest among scientists from a variety of nations [2].

3-Hydroxybutanone (Acetoin, AC) and 2,3-butanediol (BD) are two important four-carbon platform compounds with a wide range of applications in pharmaceutical and chemical synthesis [1, 3]. Synthetic chemistry, particularly drug synthesis, necessitates the optical structure and purity of compounds. The optical structure and purity of compounds are necessary for chemical synthesis, especially drug synthesis. However, since AC has two optical isomers (3R-AC and 3S-AC) and BD has three optical isomers ((2R,3R)-2,3-BD, (2S,3S)-2,3-BD, and meso-2,3-BD), the production of AC and BD by conventional chemical synthesis is typically a racemic mixture, necessitating a time-consuming and expensive purification process to obtain optically pure products. AC and BD are both overflow metabolites of the Embden-Meyerhof-Parnas (EMP) pathway of organisms, and many microorganisms can accumulate a large amount of AC and BD in the fermentation broth under high concentrations of glucose; therefore, there have been numerous studies in recent years on the production of AC and BD by microbial fermentation, and the fermentation yield has reached the level of industrial application [4]. However, it was found that AC or BD produced by microorganisms using glucose were also a mixture of their different optical isomers. AC and BD yields and the ratio of different optical isomers can vary depending on the strain and fermentation conditions; for example, the ratio of 3R-AC to 3 S-AC produced during the fermentation of *Bacillus* sp. decreases significantly with increasing fermentation time [39]; in addition, *Klebsiella pneumoniae* accumulates primarily meso-2,3-BD, accompanied by a small amount of (2R, 3R)-2,3-BD production [5]; and *Paenibacillus polymyxa* can synthesize (2R,3R)-2,3-BD with higher purity, while a small amount of meso-2,3-BD will be produced [6]. In organisms, AC is derived from α-acetolactate decarboxylation and converted to BD by butanediol dehydrogenase (BDH) in a reversible reaction. Under suitable conditions, AC and BD will be converted to each other [4, 7]. With further studies of AC and BD metabolic synthesis pathways, it was found that the production of different AC and BD optical isomers is mainly related to the specificity of BDH that catalyzes the reaction, and usually more than one BDH exists in a strain [7], which is the most important reason for the simultaneous existence of different optical isomers of AC and BD in microbial fermentation products. In this paper we first review the synthesis pathways of microbial AC and BD and the formation mechanism of various optical isomers of AC and BD, and then focus on BDH regarding its source, structure, properties and the mechanism of the production of different optical isomers of AC and BD, which provides a theoretical basis for the selection and engineering of strains for the production of high optical purity AC and BD.

## Pathways for AC and BD synthesis in microorganisms

### Metabolic pathways

**Fig. 1:**
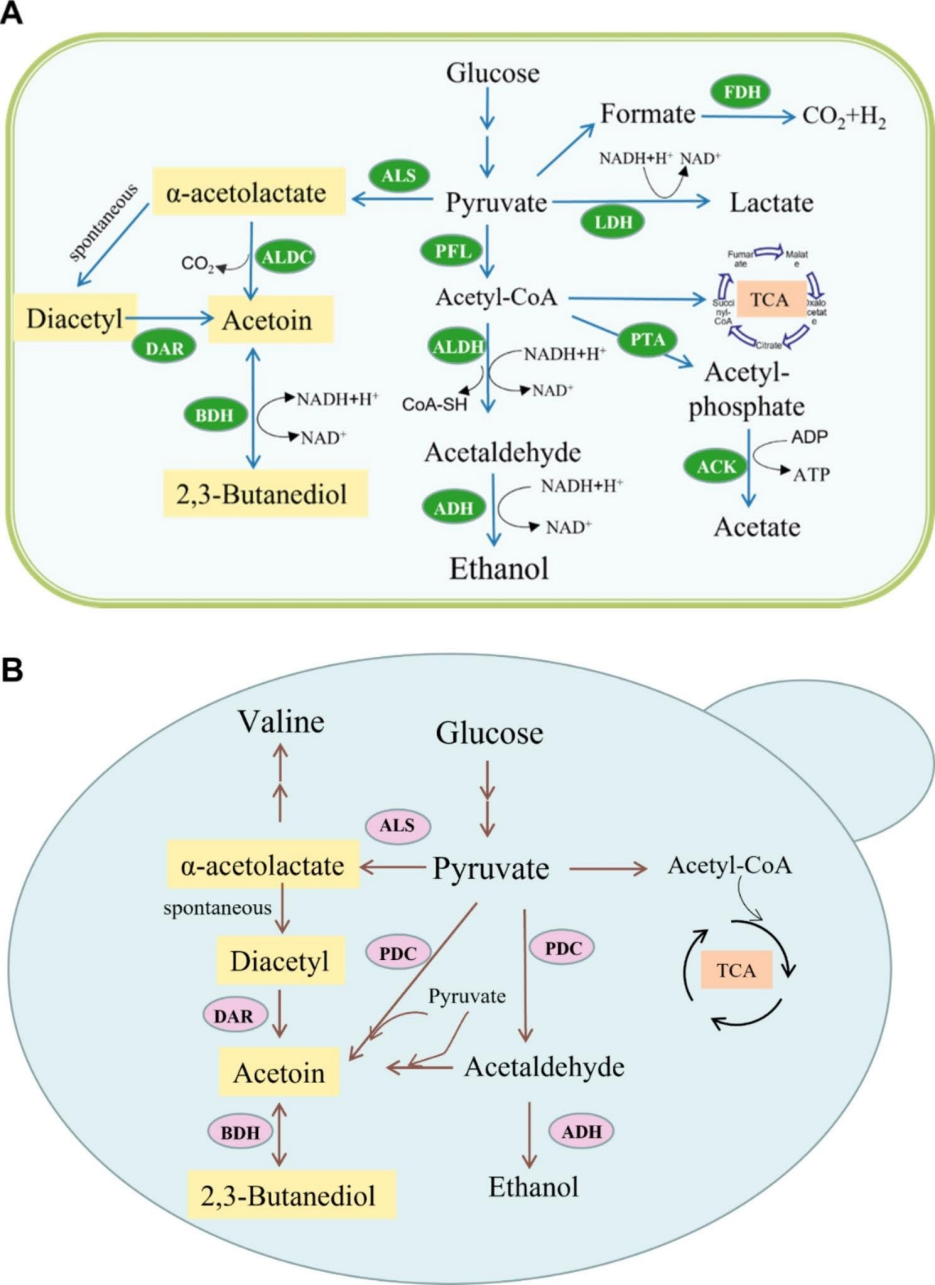
Biosynthetic pathway of 2,3-BD in 2,3-butanediol-producing bacteria (**A**) and S. cerevisiae (**B**). LDH, lactate dehydrogenase; PFL, pyruvate–formate lyase; PTA, phospho-transacetylase; ACK, acetate kinase; ALDH, acetaldehyde dehydrogenase; ADH, ethanol dehydrogenase; ALS, α-acetolactate synthase; ALDC, α-acetolactate decarboxylase; BDH, 2,3-butanediol dehydrogenase (acetoin reductase); FDH, formate-hydrogen lyase complex; DAR, Diacetyl reductase; PDC, pyruvate decarboxylase

Bacteria are currently regarded as organisms with industrial application value in AC and BD production. The main species include *Klebsiella* sp., *Bacillus* sp., *Serratia* sp., and *Pseudomonas* sp. The pathways of AC and BD synthesis in bacteria are as follows: first, pyruvate is synthesized via the EMP pathway from glucose, then AC is generated from α-acetolactate, and finally BD is produced [8]. Under anaerobic fermentation conditions, pyruvic acid is also converted into ethanol and various organic acids, such as acetic acid, lactic acid, and formic acid [9, 10] (Fig. 1A).

Three key enzymes are involved in the conversion of pyruvate to BD: α-acetolactate synthase (ALS, EC 4.1.3.18), α-acetolactate decarboxylase (ALDC, EC 4.1.1.5), and butanediol dehydrogenase (BDH, EC 1.1.1.76; also known as acetoin reductase( ACR, EC 1.1.1.4). Under slightly acidic conditions(pH 6), ALS is induced and catalyzes the formation of α-acetolactate from two molecules of pyruvate in the presence of thiamin pyrophosphate (TPP) [8]. α-acetolactate is catalyzed by ALDC to decarboxylate to AC, which tends to be reduced to 2,3-BD under oxygen-limited conditions. Meanwhile, pyruvate is converted into lactate and formate by lactate dehydrogenase (LDH, EC 1.1.1.27) and pyruvate-formate lyase (PFL, EC 2.3.1.54). With the help of the formate-hydrogen cleavage enzyme complex, formate can be broken down even further into carbon dioxide and hydrogen gas. Under oxygen-limited conditions, all enzymes and compounds involved in the AC and BD pathways in an organism are typically produced at the end of the logarithmic phase and during the stabilization phase [11]. Under aerobic conditions, the pyruvate dehydrogenase multienzyme complex preferentially catabolizes pyruvate to acetyl coenzyme A, which then enters the tricarboxylic acid cycle (TCA). Furthermore, in some microorganisms, AC may be derived from diacetyl (DA), which is produced by the catalysis of diacetyl reductase (DAR), also known as acetyl dehydrogenase (EC 1.1.1.304), and DA is commonly thought to be derived from the spontaneous decarboxylation of α-acetolactate [12–15].

In addition to bacteria, the synthetic pathways of AC and BD in *Saccharomyces cerevisiae* have been extensively studied [16] (Fig. 1B). *S. cerevisiae* does not have α-acetolactate decarboxylase. α-acetolactate cannot be directly converted to AC, but under aerobic conditions it spontaneously decarboxylates to produce DA, which is then converted to BD by BDH. Conversely, AC is made through either pyruvate or pyruvate-and-acetaldehyde condensation reactions. Generally, the flux of AC metabolism in *S. cerevisiae* is very low, and pyruvate is used for ethanol synthesis rather than AC and BD. Therefore, even though the fungus possesses synthetic pathways for AC and BD, both accumulate in negligible amounts and have no productive value.

In addition, Juni et al. proposed a BD cycle pathway in 1956 [17] (Supplementary Figure A). The cycle starts with diacetyl and goes back to diacetyl through intermediates such as acetylhydroxybutyrone (AAC), acetylhydroxybutyrone synthase (AACS), acetylhydroxybutyrone reductase (AACR), acetylbutanol hydrolase (ABDH), BDH, and DAR. However, the existence of the key enzymes in this pathway, AACS and AACR, has not been conclusively established, and the conversion of AC to DA in this pathway is widely believed to be impossible, so the BD cycle may not exist in the organism [18, 19].

### Genetic regulation of AC and BD synthesis in bacteria

**Fig. 2:**
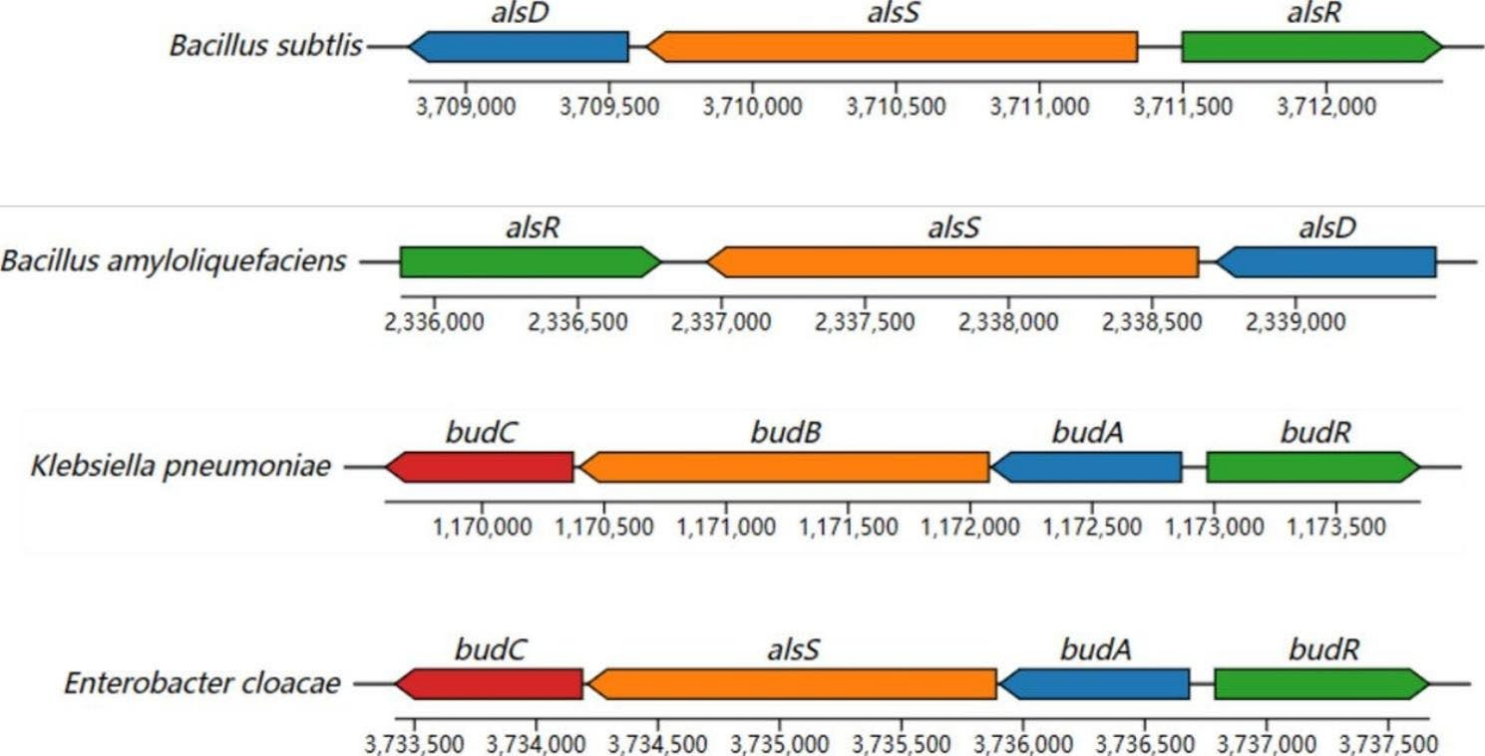
Distribution of genes encoding key enzymes of the 2,3-butanediol metabolic pathway in microorganisms. alsD, budA, gene encoding α-acetolactate decarboxylase; alsS, budB, gene encoding α-acetolactate synthase; alsR, budR, gene encoding the transcriptional activator; budC, gene encoding 2,3-butanediol dehydrogenase/acetoin (diacetyl) reductase

There are interspecies differences in AC and BD synthesis and regulation in bacteria (Fig. 2). In gram-positive bacteria such as *Bacillus* sp., the ALS and ALDC-encoding *alsS* and *alsD* genes are located on the same *alsSD* operon, while the BDH-encoding gene is located separately from the *alsSD* operon [20]. *AlsS* and *alsD* are controlled by the gene *alsR* (encoding AlsR). AlsR, a protein homologous to the LysR family of bacterial transcriptional activators, is induced by acetate, low pH, and anaerobic environments. AlsR positively regulates the transcription of the *alsSD* operon in *B. subtilis* by interacting with a cis-acting region between the *alsR* and *alsS* genes,whose deletion results in the inactivation of *alsSD* operon expression [21]. In addition, the global regulator of carbon metabolism A (Catabolite control protein A, CcpA) has been shown to participate in the regulation of the *alsSD* operon [22, 23]. In gram-negative bacteria such as *Serratia marcescens* and *Klebsiella* sp., the ALS, ALDC, and BDH-encoding genes *budB*, *budA*, and *budC* are located on the same operon *budABC* and are regulated by the regulatory gene *budR* [24, 25]. The promoter region of the *budR* gene contains an FNR-binding structural domain, and FNR is an oxygen-mediated positive regulator that directly detects intracellular oxygen concentration. FNR inhibits the expression of *budR*, resulting in BD being the main metabolite under conditions of insufficient oxygen supply to the bacterium [26]. In contrast, in *Lactococcus lactis*, although the AC and BD synthesis pathways are also present, they do not comprise an operon [27].

Additionally, quorum sensing (QS) affects the production of AC and BD in microorganisms. APHA (a population-sensing regulatory activator that activates the expression of *Vibrio cholerae* virulence genes) can bind to the upstream site of the first gene of the *alsSD* operon to repress its transcription at low cell densities [28]. In *Serratia marcescens* and *Klebsiella pneumoniae*, genes encoding ALDC and ALS were also observed to be affected by the QS system [29].

The main physiological functions of AC and BD synthesis include the prevention of excessive intracellular acidification, the regulation of the intracellular NADH/NAD^+^ ratio, and the storage of carbon and energy for microbial growth. The excessive production of acetic acid, a common metabolite of microorganisms, can result in a decrease in the pH of the environment to which the bacterium is exposed, thereby interfering with the strain’s normal growth and metabolism. To prevent acetic acid overproduction, BD-producing microorganisms induce the expression of ALS when acetic acid reaches a critical value, which makes pyruvate flow to form BD reduce the flow to acetic acid, effectively regulating the pH environment suitable for strain growth [30, 31]. In addition, in the process of sugar catabolism to produce BD, the intermediate product AC is converted to BD by BDH in a step that consumes one molecule of NADH. When glucose is depleted, NADH can be regenerated due to the reversibility of the AC reduction reaction. Therefore, it is believed that the AC and BD synthetic pathways play a role in controlling the NADH/NAD^+^ ratio in bacteria [8, 32]. In *B. subtilis*, the catabolism of AC occurs mainly through oxidative cleavage, i.e., AC is cleaved to acetaldehyde and acetyl-CoA by the action of its dehydrogenase enzyme complex system (AoDH ES), and AoDH ES is inhibited by glucose. When glucose is present, the synthetic pathway of AC or other primary metabolites is activated and the corresponding catabolic pathway is inhibited; when glucose is depleted, the inhibition is lifted and primary products such as AC and BD are consumed as energy and carbon sources to maintain cellular life mechanisms [23].

### Formation mechanism of AC and BD stereoisomers

Early studies on bacterial metabolism revealed the presence of different stereoisomers AC and BD in the organism, for which reasonable explanations have been sought [33](Supplementary Figure B). Taylor and Juni studied the mechanism of formation of AC and BD stereoisomers in *Bacillus polymyxa*, *Aerobacter aerogenes* (*K. pneumoniae*), *Pseudomonas hydrophila*, and *Bacillus subtilis* [34]. Their results led to the conclusion that all ACs from pyruvate sources are D-type (i.e., 3R-AC) and BDH is stereospecific for BD. BDH from *B. polymyxa* is predominantly of the D type, whereas BDH from *A. aerogenes* (*K. pneumoniae*) and *P. hydrophilia* is of the L type. *Bacillus subtilis* possesses both D- and L-type BDH, but this does not explain the provenance of L-AC (3S-AC). Based on the results of experiments with *P. hydrophila* cell-free extracts, they hypothesized that the organism may contain an AC racemase capable of catalyzing the conversion of 3R-AC to 3S-AC. Voloch et al. studied the mechanism of BD isomer formation in *K. pneumoniae* further in 1983 [7], purifying two AC reductases (BDH) in cell-free extracts, one converting D-AC to meso-BD and the other converting L-AC(i.e., 3S-AC) to L-BD(i.e., (2S,3S)-2,3-BD). They also discovered that cell-free extracts can change the spin values of D-AC solutions and believed the presence of AC racemase in the cytosol. In contrast, a 1984 study by Ui et al. suggested the existence of three types of BDH in *K. pneumoniae*, and their conclusion was that there are two types of meso-BDH, D-AC-forming and L-AC-forming, both of which can generate meso-2,3-BD [35]. They did not confirm the presence of AC racemase. In 1986, Ui et al. investigated the formation mechanism of different isomers of BD in *Bacillus polymyxa* and they isolated and purified these two enzymes and conducted in vitro experiments to confirm that 3S-AC could be derived from DA, effectively negating the AC racemase pathway [36].

In 1998, Ui et al. detected the enzymatic activities of the so-called AACS, AACR, and ABDH in cell-free extracts of *Bacillus cereus* YUF4 based on the BD cycling pathway [37] ( Supplementary Figure A). They believed that during the reduction of AAC by AAR, two conformations of acetylbutanediol (ABD), 3R,4R, and 3S,4R, were produced, which generated (2R,3R)-2,3-BD and meso-2,3-BD, respectively, in the presence of ABDH. Therefore, a synthetic pathway involving (2R,3R)-2,3-BD and meso-2,3-BD is proposed.

In the works on the mechanism of AC and BD stereoisomer formation, due to the technical limitations of time, the majority of the reaction properties of the enzymes were based on indirect data, and data on the reaction properties of purified enzymes were scarce. With the development of biotechnology, it has been gradually clarified that the existence of multiple BDHs in microorganisms by means of gene knockout and enzyme gene expression is the main reason for the forming of different stereoisomers AC and BD. In 2008, Nicholson et al. knocked out *bdhA*, the gene responsible for encoding R-BDH in strain *B. subtilis* 168 and discovered that a small amount of meso-2,3-BD was still detectable in the *bdhA*-deficient strain, most likely due to the presence of a second gene encoding BDH in the strain [38]. In one of our studies, a similar occurrence was observed, and we discovered that 3S-AC production was significantly reduced in *bdhA*-deficient strains, suggesting that R-BDH is involved in the synthesis of 3S-AC [39]. To date, two or more genes encoding BDH (DAR or ACR with BDH-like catalytic properties) have been identified in several bacterial strains. For example, two proteins with BDH catalytic activity were identified in *B. licheniformis*: a meso-BDH belonging to the short-chain dehydrogenase (SDR) family and a medium-chain dehydrogenase (MDR) annotated as glycerol dehydrogenase (GDH) [40]. Concurrently, both an R-BDH-encoding gene and a DAR-encoding gene were found in *Mycobacterium* sp [41]. Three BDHs (BDH1-3) and one GDH were found in *Serratia* sp. T241, and in vitro transformation tests showed that they were all involved in the creation of AC and BD isomers. BDH1 and BDH3 were crucial in BD synthesis, while BDH2 and GDH affected BD production less in *Serratia* sp. T241 [42]. In *S. cerevisiae*, in addition to BDH, arabinose dehydrogenase (ADH) was found to catalyze the production of meso-2,3-BD and (2 S,3 S)-2,3-BD from AC [43]. Furthermore, meso-2,3-BD and (2S,3S)-2,3-BD were still produced in the ADH and BDH gene double deletion strains, indicating that other enzymes with the same function are present in the strains.

**Fig. 3:**
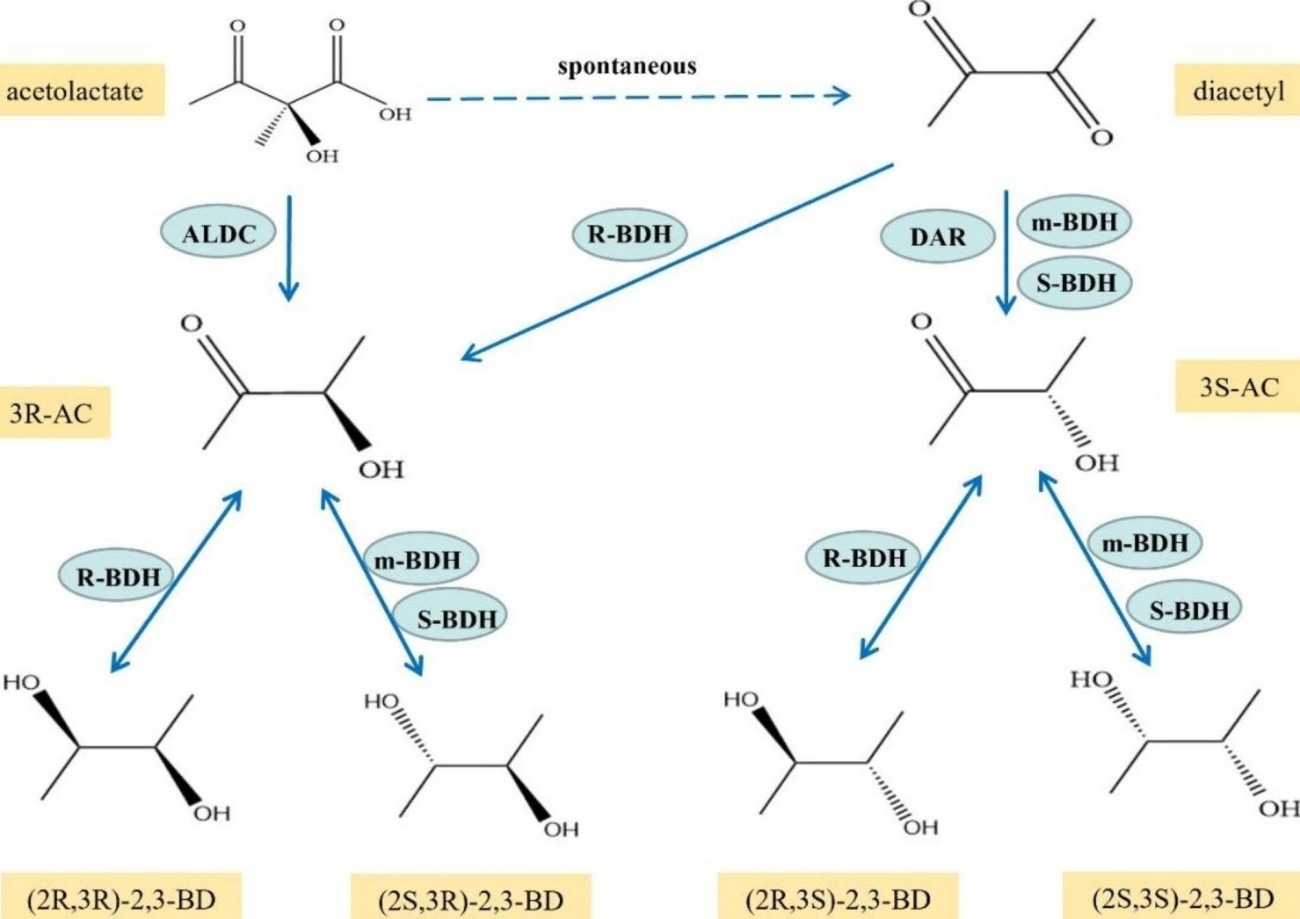
Synthesis pathways of different optical isomers AC and BD. The dotted line represents non-enzymatic oxidation. ALDC, α-acetolactate decarboxylase; DAR, Diacetyl reductase; m-BDH, meso-BDH

Combining data from the literature and databases, it was found that there are two main groups of enzymes related to the synthesis of different stereoisomers of BD, One group belongs to the medium-chain dehydrogenase (MDR) family, mainly R-BDH and aldehyde dehydrogenases (e.g., GDH), which have an R-chiral center and generally catalyze 3R-AC to produce (2R,3R)-2,3-BD and 3S-AC to produce meso-2,3-BD, mostly found in *B. subtilis*, *P. polymyxa*, *R. erythropolis*, and other gram-positive bacteria [38, 44, 45]. The other group belongs to the short-chain dehydrogenase family and contains meso-BDH, S-BDH, and DAR, which has an S-chiral center and catalyzes the production of meso-2,3-BD from 3R-AC and (2S,3S)-2,3-BD from 3 S-AC and is mainly found in *K. pneumoniae*, *S. marcescens*, and other gram-negative bacteria [46, 47]. We summarized the synthetic pathways of the various stereoisomers of AC and BD based on the most recent research results (Fig. 3). In addition to DA, 3S-AC can be synthesized from 3R-AC through a two-step conversion of meso-BDH and R-BDH (Fig. 3). From the results in the literature thus far, it appears that enzymes with BDH properties are widely present in microorganisms and are not strictly specific, and other proteins annotated as alcohol dehydrogenase enzymes may also be involved in the synthesis of different isomers of AC and BD in vivo. The next part of this article will focus on the properties and structural features of enzyme proteins with BDH properties that have been characterized and have been clearly annotated.

## Properties, structure, and substrate specificity of BDH (ACR, DAR)

### Physicochemical properties and specificity of the substrate

#### R-BDH

Currently, R-BDH derived from *P. polymyxa*, *R. erythropolis*, *Bacillus* sp., and *Bacillus thuringiensis* has been characterized [44, 45, 48, 49] (Table 1). The characterized R-BDH has a molecular weight of approximately 37 kDa and is usually composed of two or four subunits. R-BDH has both oxidative and reductive activities and uses NAD(H) as a coenzyme. In vitro experiments show that the optimal pH for its oxidative and reductive activities is not the same. The optimal pH for reduction activity is 6–7, while the optimal pH for oxidation activity is 8–10. In vitro catalysis under physiological conditions (pH 7.0-7.5) showed that its reductase activity was much greater than its oxidase activity, preferring to convert AC to BD, but in organisms its redox activity was mainly dependent on NAD(H) concentration and less influenced by pH, especially its oxidative activity. Unlike the pH, the optimum temperature for the R-BDH reaction is mainly related to the source of the strain. In *P. polymyxa* ZJ-9, the optimal temperature for R-BDH reduction activity was 30 °C, while the optimal temperature for oxidation activity was up to 80 °C. The optimal temperature for the R-BDH reduction activity of *R. erythropolis* WZ010 was 55 °C, and the optimal temperature for oxidation activity was 45 °C. The optimal temperatures for the reducing and oxidizing activities of BDH derived from *B. thuringiensis* are 35 and 50 °C, respectively. In vitro reactions show that EDTA inhibits R-BDH activity, indicating that the enzyme reaction requires the participation of metal ions. The stability of R-BDH derived from various strains varied considerably. R-BDH from *P. polymyxa* ZJ-9 remained stable at temperatures between 20 and 30 °C, but its stability rapidly decreased at temperatures above 40 °C [44]. R-BDH derived from *B. thuringiensis* was stable over a broad pH range (6–10) and at temperatures up to 70 °C, with excellent stability after three months of storage at 4 °C [49]. In vitro reactions the most suitable substrates of R-BDH were (2R,3R)-2,3-BD, meso-2,3-BD, and 3R/3S-AC. Furthermore, R-BDH can catalyze the dehydrogenation of glycerol, 2-propanol, 1,2-propanediol, 1,2-pentanol and glyceraldehyde-3-phosphate [50].

**Table 1:** The physicochemical properties and substrate specificity of the characterized R-BDH

Strains	Substrate	Product	K_m_	Optimal pH	Optimal temperatures	Reference
*Bacillus sp*. DL01	DA	3R-AC	0.626 ± 0.12	7.4	40	(1)
	3R/3S-AC	(2R,3R)/meso-2,3-BD	0.253 ± 0.003	5.5	35	
	meso-2,3-BD	3S-AC	0.397 ± 0.11	7.4 ~ 8	40	
	(2R,3R)-2,3-BD	3R-AC	0.389 ± 0.090			
*R. erythropolis* WZ010	DA	3R-AC	0.1 ± 0.001	ND	55	(2)
	3R/3S-AC	(2R,3R)/meso-2,3-BD	ND	6.5		
	meso-2,3-BD	3S-AC	ND	10	45	
	(2R,3R)-2,3-BD	3R-AC	0.58 ± 0.05			
*P. polymyxa* ZJ-9	3R-AC	(2R,3R)-2,3-BD	0.20 ± 0.02	6.0	30	(3)
	3S-AC	meso-2,3-BD	0.84 ± 0.05			
	meso-2,3-BD	3S-AC	2.73 ± 0.02	8.0		
	(2R,3R)-2,3-BD	3R-AC	7.67 ± 0.73			
*B. thuringiensis*	DA	3R-AC	0.66 ± 0.06	7.5	35	(4)
	3R/3S-AC	(2R,3R)/meso-2,3-BD	0.49 ± 0.08			
	meso-2,3-BD	3S-AC	0.76 ± 0.12	10	50	
	(2R,3R)-2,3-BD	3R-AC	3.67 ± 0.55			

In addition to classical R-BDH, it has been discovered that the accumulation of (2R,3R)-2,3-BD in *K. pneumoniae* is closely related to GDH [5]. The external conversion shows that GDH can convert 3R-AC/3S-AC into (2R,3R)-2,3-BD and meso-2,3-BD, respectively.

#### meso-BDH

The characterized meso-BDHs come from *K. pneumoniae*, *B. licheniformis*, and *S. marcescens* [46, 47, 51] (Table 2). Meso-BDH has a molecular weight of approximately 30 kDa and is usually made up of two or four subunits. Similar to R-BDH, the optimal pH of reductase activity and oxidase activity of meso-BDH was inconsistent; the optimum pH of reductase activity was slightly lower than that of R-BDH, and the optimum pH of oxidase activity was 8–10. However, there were strain differences; for instance, the optimum pH of reductase activity of meso-BDH from *K. pneumoniae* XJ-Li was rarely as high as 8.0. The optimum reaction temperatures for the two enzyme activities, however, were the same. For instance, the optimum reaction temperature for both the reducing and oxidizing activities of meso-BDH in *S. marcescens* H30 was 40 °C, the optimum reaction temperature for both enzyme activities of meso-BDH in *K. pneumoniae* XJ-Li was 35 °C, and meso-BDH in *B. licheniformis* was 37 °C. The meso-BDH from *K. pneumoniae* exhibited high stereospecificity with high affinity for 3R-AC/3S-AC, which rapidly reduced racemic AC to meso-BD and (2S,3S)-2,3-BD, and showed no significant activity against (2R,3R)-2,3-BD, 1,4-butanediol and (2S,3S)-2,3-butanediol [47].

**Table 2:** The physicochemical properties and substrate specificity of the characterized R-BDH

Strains	Substrate	Product	K_m_	Optimal pH	Optimal temperatures	Reference
*K. pneumoniae* XJ-Li	DA	3S-AC	ND	ND	ND	(5)
	3R/3S-AC	meso/(2 S,3 S)-2,3-BD	0.65 ± 0.11	8.0	35	
	meso-2,3-BD	3R-AC	13 ± 0.32	9.0		
	(2S,3S)-2,3-BD	3S-AC	ND			
*S. marcescens* H30	DA	3S-AC	3.3 ± 0.1	8.0	ND	(6)
	3R/3S-AC	meso/(2 S,3 S)-2,3-BD	0.97 ± 0.08	5.0	40	
	meso-2,3-BD	3R-AC	4.1 ± 0.3	8.0		
	(2S,3S)-2,3-BD	3S-AC	31.2 ± 0.4	ND		
*B. licheniformis*	DA	3S-AC	72.4 ± 0.4	5.0	ND	(7)
	3R/3S-AC	meso/(2S,3S)-2,3-BD	0.47 ± 0.03		37	
	meso-2,3-BD	3R-AC	7.25 ± 0.05	10.0		
	(2S,3S)-2,3-BD	3S-AC				

#### S-BDH

Only BDH from *Brevibacterium saccharolyticum* C-1012 (later renamed *Corynebacterium glutamicum*), which was the only strain confirmed to have S-BDH activity, was characterized in detail [52]. The enzyme has four subunits and a molecular weight of approximately 30 kDa. The optimum pH values for reduction and oxidation activities were 6.0 and 10.5, respectively. Acetic acid could inhibit its activity. The enzyme had a greater affinity for (2S,3S)-2,3-BD than for 3S-AC and no discernible activity for meso-2,3-BD or (2R,3R)-2,3-BD. The km values for 3S-AC and (2S,3S)-2,3-BD were 0.23 ± 0.02 and 0.052 ± 0.005 mM, which were lower than those for 3R-AC and meso-2,3- BD of 1.49 ± 0.07 and 2.01 ± 0.40.

#### DAR

Wang et al. purified *R. erythropolis* WZ010 DAR (ReADR) [50]. This enzyme is a homodimer consisting of 26 kDa subunits. The optimal pH and temperature for ReADR’s reducing activity are 7.0 and 30 °C, whereas the optimal pH and temperature for its oxidizing activity are 9.5 and 25 °C. Under optimized conditions, the enzyme’s activity for the reduction of DA was 11.9 times greater than the oxidation activity of (2S,3S)-2,3-BD. The kinetic parameters of the enzyme showed lower km values and higher catalytic efficiency for DA and NADH compared to (2S,3S)-2,3-BD, and NAD^+^. The enzyme accepts a variety of substrates, such as fatty and aromatic alcohols, aldehydes, and ketones. One DAR from *B. polymyxa* IAM 1189 has the highest activity at pH 5.8 to 6.0 and is stable below 35 °C but rapidly deactivates above 50 °C [53]. Giovannini et al. identified an NADH-dependent DAR (ACR) from *B. searothermophilus* that is also a homodimer and catalyzes the reduction of DA to 3S-AC, followed by (2S,3S)-2,3-BD in the presence of NADH; in the presence of NAD^+^, it is able to catalyze the oxidation of (2S,3S)-2,3-BD and meso-BD to 3S-AC and 3R-AC, but not to DA [54]. Additionally, the enzyme also catalyzes redox reactions involving endocyclic bicyclooctenes, heptenols, and their respective ketones.

Thus far the BDHs that have been thoroughly characterized are relatively limited, and their substrate and product stereoisomeric properties are derived primarily from in vitro experiments. Most BDHs are multi-substrate and may have a higher affinity for a specific substrate. Compared to R-BDH and meso-BDH, the sources and properties of S-BDH and DAR have been less studied, especially DAR, which belongs to SDR like S-BDH and has substrate product properties similar to those of S-BDH. However, current studies are not yet able to effectively distinguish between them, and more data are needed to support this.

### BDH structural features

**Fig. 4:**
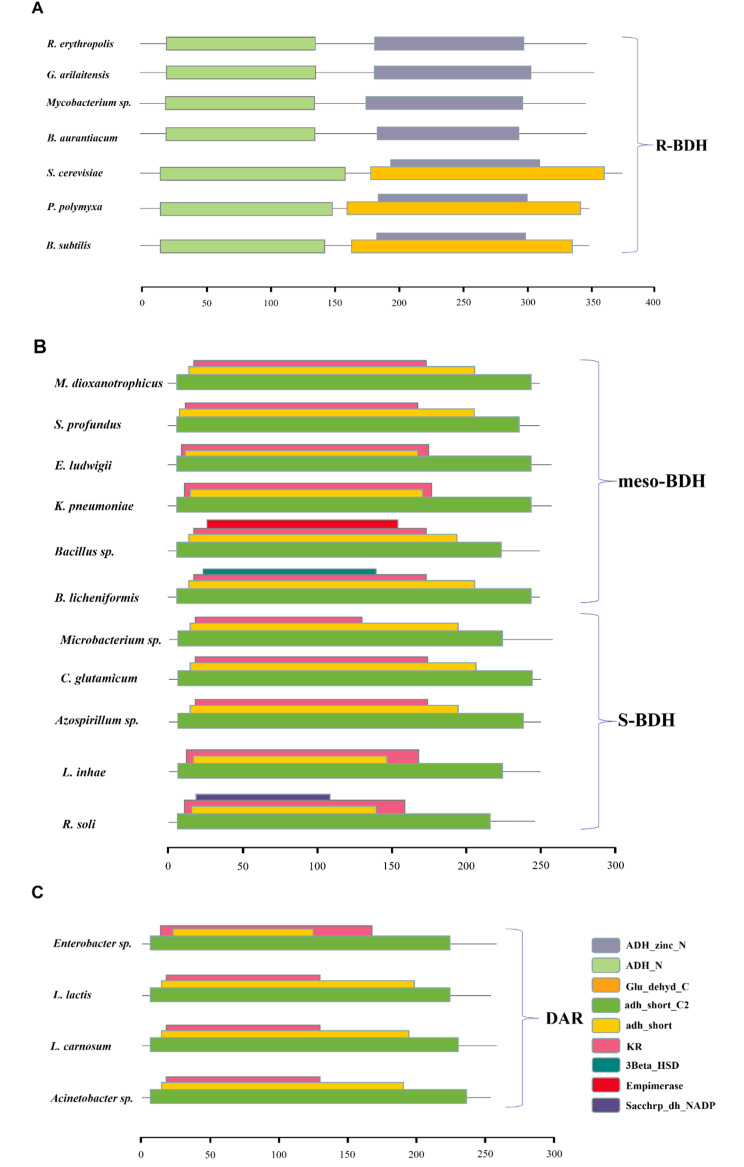
Conserved domains of BDHs and DARs. The selected amino acid sequences were derived from UniProt, GenBank, and KEGG databases, and conserved domain alignment was performed using the PFAM network tool. The sequence accession number and PFAM ID are as follows: R. erythropolis, UniProt ID A0A0E4A9D6; G. arilaitensis, UniProt ID E1W0D5; Mycobacterium sp., UniProt ID F1T242; B. aurantiacum, UniProt ID A0A2A3YZ33; S. cerevisiae, UniProt ID P39714; P. polymyxa, UniProt ID E7EKB8; B. subtilis, UniProt ID O34788; M. dioxanotrophicus, KEGG ID BTO20_28760; S. profundus, UniProt ID A0A1U7D589; E. ludwigii, KEGG ID EcWSU1_01150; K. pneumoniae, GenBank ID AFB82681.1; Bacillus sp., GenBank ID QIS93452.1; B. licheniformis, KEGG ID BLi02066;Microbacterium sp., UniProt ID A0A0F2C224; C. glutamicum, UniProt ID Q9ZNN8; Azospirillum sp., UniProt ID A0A839W257; L. inhae, UniProt ID A0A175D337; R. soli, UniProt ID A0A7 × 0MQE3; Enterobacter sp., UniProt ID V3PYV0; L. lactis, UniProt ID Q9RLV7; L. carnosum, UniProt ID K0D762; Acinetobacter sp., UniProt ID L9LU15. ADH_zinc_N PFAM ID PF00107, ADH_N PFAM ID PF08240, Glu_dehyd_C PFAM ID PF16912, adh_short_C2 PFAM ID PF13561.9, adh_short PFAM ID PF00106.28, KR PFAM ID PF08659.13, Sacchrp_dh_NADP PFAM ID PF03435.21, 3Beta_HSD PFAM ID PF01073.22, Epimerase PFAM ID PF01370.24 (**A**) The conserved domain of R-BDH. (**B**) The conserved domain of meso-BDH and S-BDH. (**C**) The conserved domain of DAR

**Fig. 5:**
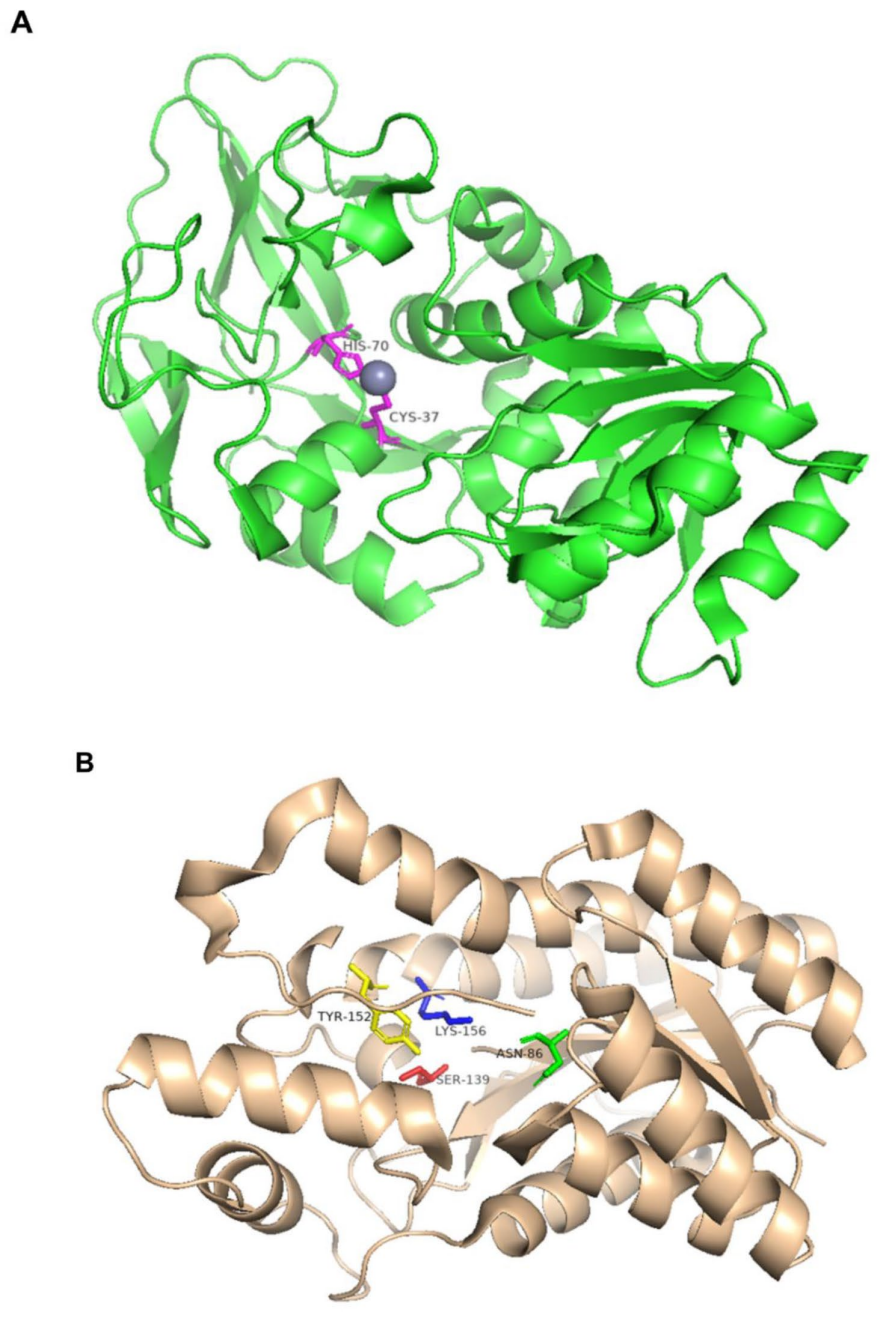
The structural information of the protein is derived from the PDB database and visualized using pymol. (**A**) The catalytic zinc binding site of R-BDH in B. subtilis 168 (PDB ID 6IE0). (**B**) Catalytic tetrad of meso-BDH in K. pneumoniae IAM 1063 (PDB ID 1GEG)

To better understand the specificity of the substrate and product for BDH, we searched the protein amino acid sequence information of several types of BDH (including DAR) from different sources in databases such as PFAM and CDD and analyzed and compared the structural domains present in those BDHs by web tools and software. BDH is typically dimeric or tetrameric, and based on the comparison results, it is evident that the R-BDH single chain encodes an amino acid sequence of approximately 350 aa and has two conserved structural domains(Fig. 4A): the ethanol dehydrogenase GroES-like structural domain (ADH_N, PFAM ID PF08240) and the zinc-binding dehydrogenase structural domain (ADH_zinc_N,PFAM ID PF00107), which is consistent with the analysis of Yu et al [6]. It is worth noting that a glucose dehydrogenase C-terminal sequence (Glu_dehyd_C, PFAM ID PF16912) overlapping with ADH_zinc_N was also found in the R-BDH sequences of *S. cerevisiae* S288C, *Bacillus* sp. DL01, and *B. subtilis* 168, a sequence normally found in glucose 1-dehydrogenase (EC1.1.1.47), which catalyzes the NAD(P)(+)-dependent oxidation of D-glucose to D-gluconate. This suggests that this enzyme may also have glucose dehydrogenase properties. The N-terminal region of the peptide chain has an all β-fold catalytic structural domain; each subunit has two tightly bound zinc atoms, with catalytic zinc in the active site, coordinated by histidine and cysteine residues and water molecules, and structural zinc in the catalytic structural domain, which acts as structural support and influences intersubunit interactions. In addition, two hydrophobic residues, Phe and Leu, comprise the coenzyme binding site, which is a conserved residue for the majority of medium-chain dehydrogenases (Fig. 5A).

Both S-BDH and meso-BDH belong to the NAD(H)-dependent short-chain dehydrogenase family (SDR), whose single chain encodes amino acids approximately 250 aa in length, and sequence analysis revealed that they both have a short-chain dehydrogenase structural domain (adh_short, PFAM ID PF00106.28), an enoyl-(acyl carrier protein) reductase structural domain (adh_short_C2, PFAM ID PF13561.9) and a ketoacyl reductase domain (KR, PFAM ID PF08659.13), which is also part of the bacterial polyketide synthase (Fig. 4B). In addition, a variety of other protein structural domains overlapping with the abovementioned structural domains were identified. For instance, the Sacchrp_dh_NADP binding domain (PFAM ID: PF03435.21) is found as a bifunctional polypeptide with lysine ketoglutarate reductase in some organisms. The 3Beta HSD (PFAM ID: PF01073.22) structural domain catalyzes the oxidation and isomerization of 5-ene-3-beta-hydroxypregnene and 5-ene-hydroxyandrostene steroid precursors into the corresponding 4-ene-ketosteroids necessary for the formation of all classes of steroid hormones. The epimerase (PFAM ID: PF01370.24) structural domain is a family of proteins that utilize nucleotide-sugars as substrates for a variety of chemical reactions. These structural domains are nonspecific and do not exist alone in specific enzymes, and these characteristics of S-BDH and meso-BDH point to a broad and nonspecific origin for BDH.

**Fig. 6:**
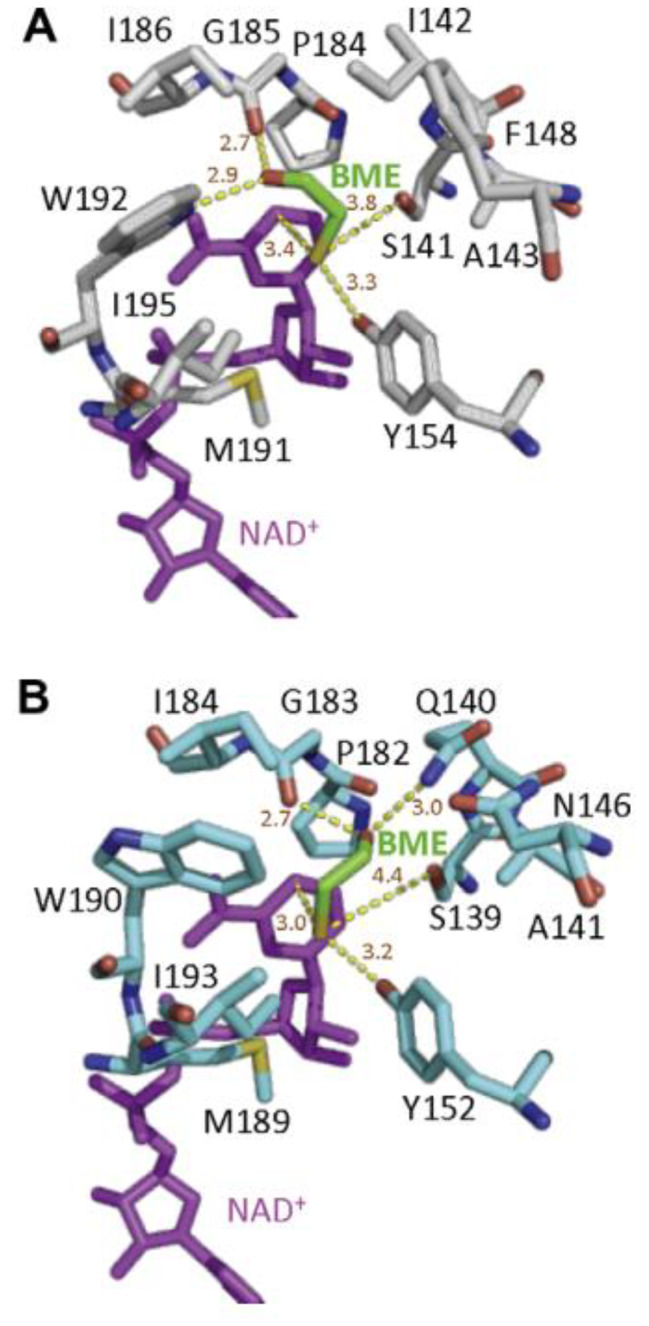
Close-up views of the mercaptoethanol-binding site in S-BDH (**A**) and in meso-BDH (**B**). Hydrogen bond distances and the distances from catalytic residues are depicted as yellow broken lines [54]

A common “Asn-Ser-Tyr-Lys” catalytic tetramer was identified in the SDR family based on biochemical and crystallographic analyses. In the S-BDH and meso-BDH crystal structures, conserved active site residues of Asn_86_, Ser_139_, Tyr_152_, and Lys_156_ were identified near the NAD^+^ molecule (Fig. 5B), which is consistent with the catalytic tetramer structure in the SDR family [55]. Meso-BDH and S-BDH share a similar overall structure and coenzyme-binding mechanism but have distinct substrate preferences. Otagiri et al. compared the mode of substrate binding between S-BDH derived from *C. glutamicum* and meso-BDH from *K. pneumoniae* [55]. They observed the binding mode of mercaptoethanol (ME) in the spatial structure of the two BDHs using competitive inhibition of ME and discovered that the bound ME was in roughly the same position in S-BDH as in meso-BDH, with the same hydrogen bonds derived from Tyr residues and Ser residues with catalytic functions, both very close to ME. The difference between the two binding modes is that the hydroxyl group of ME in S-BDH is hydrogen bonded to Gly_185_ and Trp_192_, whereas it is linked to Gly_183_ and Gln_140_ in meso-BDH (Fig. 6). Additionally, two amino acid residues in the active centers of the two enzymes were different (Ile142, and Phe148 in S-BDH versus Gly140, and Asn146 in meso-BDH). They attempted to alter their substrate preference by exchanging two residues in the active sites of S-BDH and meso-BDH but were unsuccessful. This result indicates that the stereoselectivity of BDH is not solely dependent on the active center, but it is still unknown which factors underlie the substrate’s affinity.

DAR is also a member of SDR, which are typically tetramers with a single chain encoding approximately 250 aa of amino acids. The 3D structures of the conserved structural domains of DAR are comparable to those of S-BDH and meso-BDH, including adh_short_ C2, adh_short, and KR (Fig. 4C). Because of the absence of information about the stereo structure of DAR, it was not possible to compare the active site of DAR with S-BDH and meso-BDH.

### Homology analysis of BDH

**Fig. 7:**
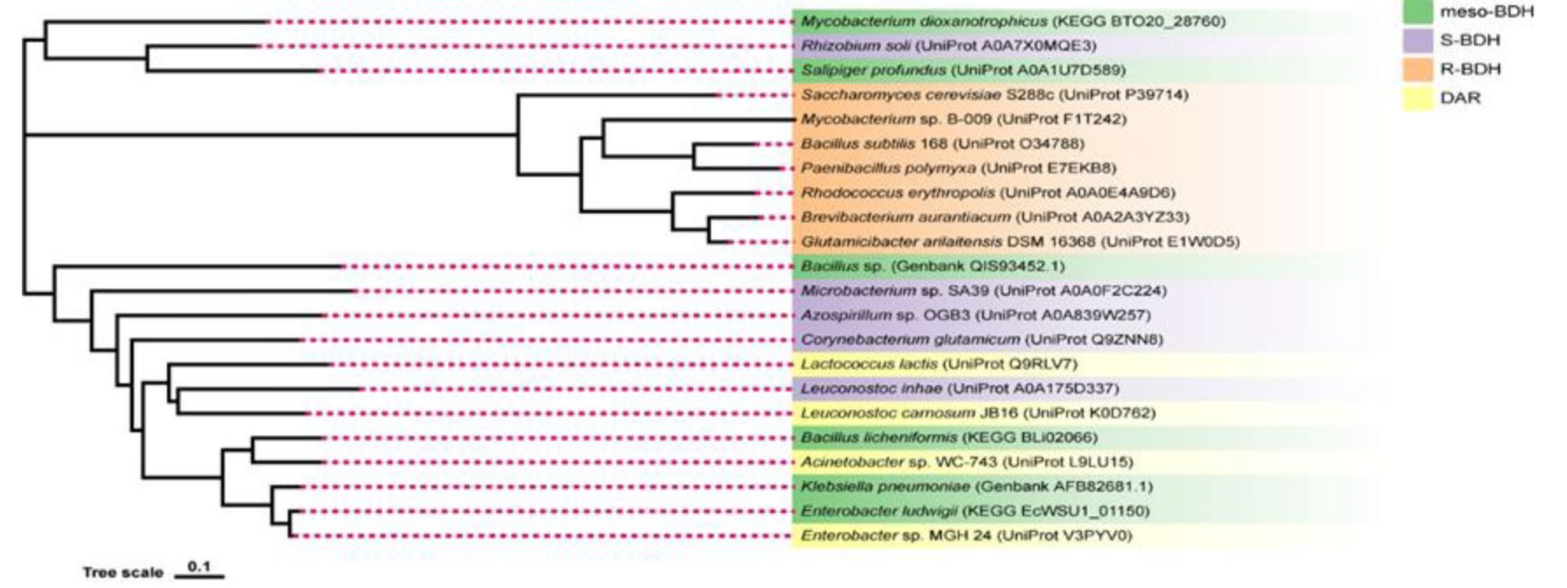
Phylogenetic tree of BDHs and DARs

We chose BDH and DAR sequences from BREND, UniProt, KEGG, GenBank, and other databases to construct a phylogenetic tree (Fig. 7) to investigate the homology between BDH and DAR. The results showed that the R-BDHs from different bacterial species were all in the same main branch, and the R-BDHs from *S. cerevisiae* and the R-BDHs from bacteria were divided into different subbranches, indicating that the R-BDHs from different sources were relatively close. Meso-BDH, S-BDH and DAR from different species are divided into two main branches and each type is dispersed in different subbranches, indicating that there may be inconsistencies between the current annotation and naming of these sequences and their structural and catalytic properties and that more research on the catalytic properties of these enzyme proteins is required to facilitate their more accurate classification.

**Fig. 8:**
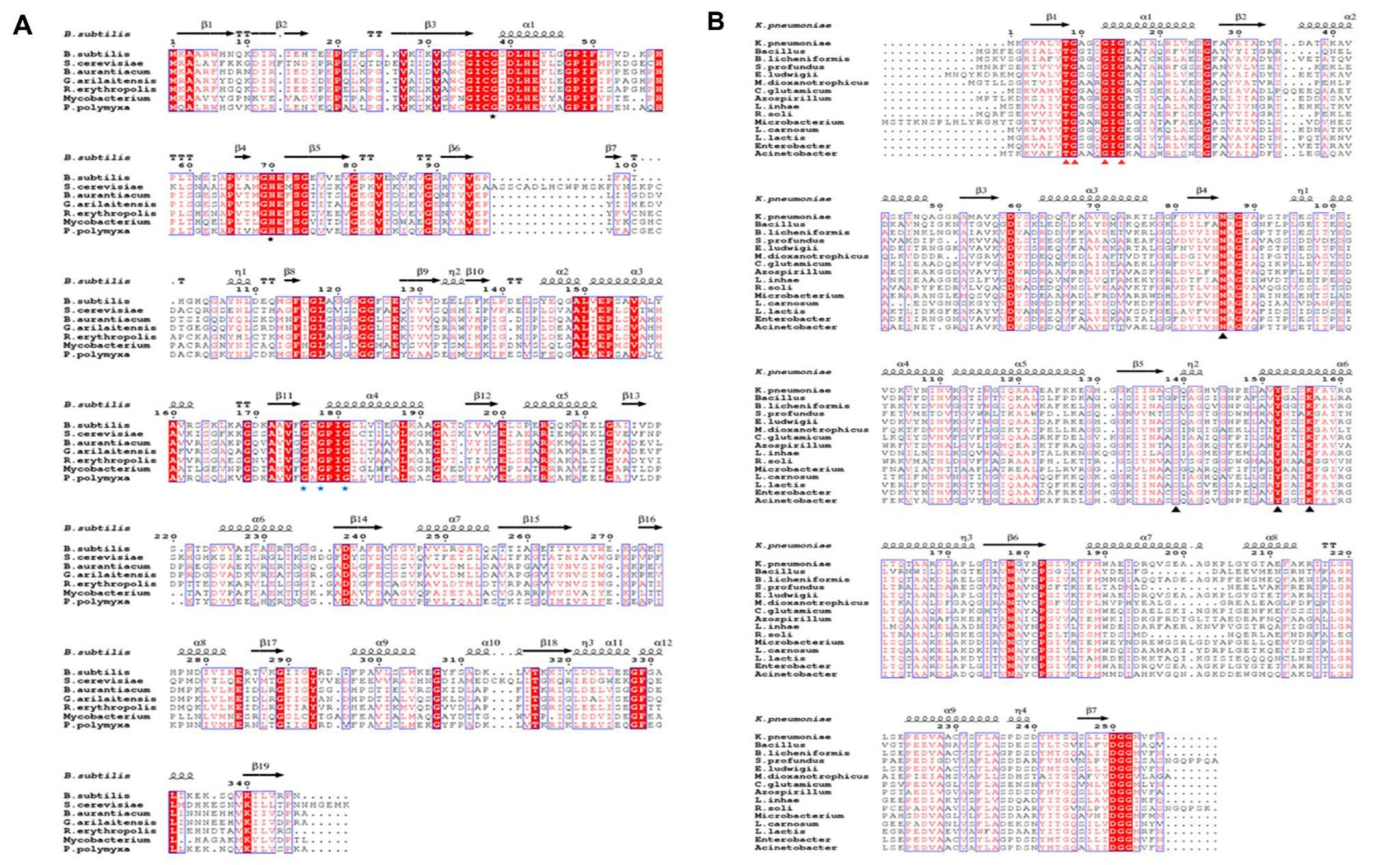
Structure-related sequence alignment between BDH and DAR. The amino acid sequence was derived from UniProt, GenBank, and KEGG databases. The sequence structure information was derived from the PDB database and visualized using the ESPript 3 network tool. (**A**) Structure-related sequence alignment between R-BDH. The black pentagram is the binding site of catalytic zinc. The blue pentagram is a coenzyme-binding motif. (**B**) Structure-related sequence alignment among S-BDH, meso-BDH and DAR. The red triangle is a coenzyme binding motif. The black triangle is the active center motif

Based on the phylogenetic tree, all R-BDHs were grouped into one group, meso-BDH, S-BDH and DAR were grouped into another group, and their protein sequence homology was compared (Fig. 8). The results revealed that all R-BDH sequences contained the catalytic zinc coordination residues Cys_37_ and His_70_, as well as a segment of the coenzyme-binding motif G_176_XG_178_XXG_181_ [56]. The sequence comparison of meso-BDH, S-BDH, and DAR showed a coenzyme-binding motif (T_9_G_10_XXXG_14_XG_16_) at the turn between the first β-strand and the second α-helix, and an active center motif (Y_152_XXXK_156_) at the C-terminus, consistent with the conserved catalytic tetrad (Asn_86_, Ser_139_, Tyr_152_, Lys_156_), except for the meso-BDH amino acid sequence derived from *Bacillus* sp. where position 139 is not Ser but Pro.

A comparison of the amino acid sequence identity and similarity of enzyme proteins revealed that the *B. subtilis* R-BDH amino acid sequence shared greater than 80% sequence similarity with *P. polymyxa*, while the other species shared between 50 and 60% sequence similarity (Supplementary Table A.1). The sequence of meso-BDH from *K. pneumoniae* was even more similar to several DARs than to some meso-BDHs, and the DAR from *Enterobacter* sp. MGH 24 was more than 90% similar to it (Supplementary Table A.2). Moreover, the consistency and similarity of the amino acid sequences of *C. glutamicum*-derived S-BDH with several other S-BDHs and DARs revealed no significant differences between DARs and S-BDHs (Supplementary Table A.3). Consequently, the DAR annotated in the current database may also be a meso-BDH or S-BDH.

Currently, BDH nomenclature is largely dependent on the enzyme’s substrates and f products; there is no strictly uniform naming system, and S-BDH and meso-BDH have nearly identical functions. Notably, the annotation of meso-BDH in the KEGG and UniProt databases is also ambiguous, and the majority of meso-BDHs are concurrently and generically named S-BDH. To determine the nature of a particular sequence, therefore, confirmation of the catalytic properties of an enzyme protein is still necessary.

## Production of optically pure AC/BD

### Screening of wild strains

Most bacteria have more than one type of BDH and so their products are usually a mixture of various optical isomers of AC or BD, but there are also some microorganisms in nature that can produce optically pure AC or BD. Dai et al. isolated one *B. subtilis* strain from marine sediments and obtained 83.7 g/L of (3R)-AC with an optical purity of 99.4% using glucose as the carbon source [57]. Zhong et al. screened a thermophilic strain H-18 W from seven strains of *Bacillus* sp., which produced 21.84 g/L (3R)-AC with an optical purity of 96.25% under fermentation conditions of 45 °C. Diacetyl is a natural precursor of 3S-AC in bacterial metabolism, and it can only be generated through the nonenzymatic oxidation of α-acetolactate with very low accumulation. Therefore, direct fermentation to produce 3S-AC is extremely challenging, and no strain capable of producing it naturally has been identified.

The ability of a variety of bacteria to produce BD has been studied [4], but only *P. polymyxobacteria* and *B. subtilis* have been found to produce (2R,3R)-2,3-BD with an optical purity exceeding 98% [6, 58]. To date, there has been no report about wild microorganisms producing optically pure meso-2,3-BD and (2S,3S)-2,3-BD.

### Editing of wild strains

It is evident that microorganisms simultaneously produce various mixtures of AC and BD, primarily because the strains have more than one type of BDH. Therefore, gene knockout and overexpression can increase or decrease the production of a particular stereoisomer for the production of optically pure AC or BD (Table 3).

**Table 3:** Optically pure AC and BD were obtained by editing wild strains

Strains	Methods	Product	Enantiomeric excess (%)	Reference
*S. marcescens* MG1	Deletion of *slaC* and *gidA*	3R-AC	/	(8)
*K. pneumoniae*	Deletion of *budC* and *acoABCD*	3R-AC	96	(9)
*C. glutamicum* CGR6	Block the reduction of AC to BD, weaken the TCA cycle and insert more *alsSD* operon copies into the host genome	3R-AC	95	(10)
*L. lactis*	Deletion of *ldh, ldhB*, *ldhX*, *pta*, *adhE*, and *butBA*; and overexpression of the lactose-utilization pathway	3S-AC	/	(11)
*S. marcescens*	Deletion of *slaC* and overexpression of *bdhA*	(2R,3R)-2,3-BD	/	(12)
*Enterobacter cloacae* sp.	Deletion of *ptsG*, *ldh*, *frdA*, *adh* and overexpression of Galp	(2R,3R)-2,3-BD	> 97.5	(13)
*P. polymyxa* ZJ-9	Deletion of *DudA*	(2R,3R)-2,3-BD	99	(14)
*K. oxytoca ΔldhA ΔpflB*	Deletion of *budC* and expression of *bdhA* from *P. polymyxa*	(2R,3R)-2,3-BD	92	(15)
*S. cerevisiae*	Deletion of PDC and overexpression of MTH1, cytoILV2, ALSD and BDH	(2R,3R)-2,3-BD	100	(16)
*B. licheniformis* Δ*budC*	Deletion of *budC*	(2R,3R)-2,3-BD	99.4	(17)
*B. licheniformis* Δ*gdh*	Deletion of *gdh*	meso2,3-BD	99.2%	(17)
*B. subtilis*	Deletion of *bdhA, acoA, pta, Idh*, and overexpression of *budC, alsS, alsD*	meso2,3-BD	100	(18)

### 3R-AC

Bacteria with *alsSD* operons produce 3R-AC via acetolactate decarboxylation; therefore, theoretically only the knockout of multiple BDHs in wild strains is necessary to achieve high optical purity in 3R-AC. For instance, Lv et al. knocked out the meso-BDH and GDH-encoding genes *slaC* and *gidA* in *S. marcescens* MG1 and prevented the conversion of AC to BD, obtaining strains with the ability to accumulate large amounts of 3R-AC [59]. Wang et al. eliminated the *budC* gene encoding meso-BDH in *K. pneumoniae* CGMCC 1.6366 and obtained 3R-AC with a 96.0% optical purity [60]. To further increase the 3R-AC yield, they employed overexpression of transcriptional regulatory genes or deletion of the assimilated AC dehydrogenase line acoABCD responsible for AC, respectively.

### (2R,3R)-2,3-BD/meso-2,3-BD

In the process of gene editing wild strains to produce optically pure BD, the deletion or introduction of various BDH-encoding genes is the primary method. For instance, Bai et al. deleted the meso-BDH gene from *S. marcescens* and introduced the R-BDH gene from *B. subtilis* and the recombinant strain was able to produce 89.81 g/L (2R, 3R)-2,3-BD in 48 h of batch replenishment fermentation [61]. Zhang et al. eliminated the DAR gene (*DudA*) in *P. polymyxa* ZJ-9 through single-hybrid homologous recombination, and (2R,3R) -2,3-BD comprised 99.9% of the fermentation broth [62]. 90% of the BD produced by *K. oxytoca* was meso-BDH. Park et al. deleted *budC*(meso-BDH gene) in one *K. oxytoca* strain and expressed R-BDH from *P. polymyxa* [63]and the modified strain yielded 106.7 g/L(2R,3R)-2,3-BD with 92% optical purity. *Bacillus licheniformis* contains two genes involved in BD synthesis: *budC* and *gdh*( encoding GDH). By deleting *budC* or *gdh*, Ge et al. created two efficient mutant strains that were used to produce meso2,3-BD (99.2% purity) and (2R,3R)-2,3-BD (99.4% purity), respectively [64]. *B. subtilis* is a common AC-producing strain, and the product contains a trace amount of BD, primarily (2R,3R)-2,3-BD and a trace amount of meso-2,3-BD. Fu et al. deleted the *bdhA*, *acoA*(AC dehydrogenase enzyme system coding gene), *pta*(phosphotransacetylase coding gene), and *ldh*(lactate dehydrogenase coding gene ) of *B. subtilis* to prevent the production of (2R,3R)-2,3-BD, acetate, lactate, and degradation of AC [65] and then introduced the *budC* gene from *K. pneumoniae* to construct a strain of *B. subtilis* that can produce pure meso-2,3-BD.

### 3S-AC, (2S,3S)-2,3-BD

There was no scientific explanation for the 3S-AC for a very long time. Initially, it was believed that a racemase catalyzed the conversion of 3R-AC and 3S-AC, but this enzyme has yet to be identified. Later, it was found that α-acetyllactate could be oxidized nonenzymatically to produce DA, which was catalyzed by DAR to produce 3S-AC. However, the production of DA by nonenzymatic oxidation is usually very small. *Lactococcus lactis* produces more DA than other AC- and BD-producing bacteria. Liu et al. obtained the first high-yield DA by fermenting *Lactococcus lactis* using metabolic model simulations on a genome-scale and the addition of metal ions [66]. On this basis, they deleted 3R-AC as well as the lactate, acetate, and ethanol synthesis pathways and introduced two BDH genes from the *Lactococcus lactis* strain and the DAR (S-BDH) gene of *E. cloacae* and successfully constructed the 3S-AC synthesis pathway with a glucose fermentation yield of 5.8 g/L of 3S-AC. Consequently, an efficient (2S,3S)-2,3-BD synthetic pathway was constructed in *Lactococcus lactis* by introducing BDH and DAR from *E. cloacae* and *K. pneumoniae*, respectively [67].

### Pathway construction in common chassis strains

Common chassis strains, such as *E. coli*, *S. cerevisiae*, and *C. glutamicum*, have a clearer genetic background, more mature gene editing technology, and more efficient genetic manipulation than wild strains. In addition, these hosts typically lack the complete pathway for AC and BD synthesis, or the activity of the pathway is low, which provides a favorable genetic background for the synthesis of optically pure isoforms of AC or BD (Table 4).

**Table 4:** Optically pure AC and BD were obtained by heterologous expression

Strains	Methods	Product	Enantiomeric excess (%)	Reference
*E. coli* DH5α	Expression of *budRAB* and *nox* in *E. coli* DH5α	3R-AC	97.3	(19)
*C. glutamicum*	Deletion of *budC, pta, ack, ldh, nagD*, and overexpression of *alsS, alsD* in *C. glutamicum*	3R-AC	> 90	(20)
*Pichia pastoris*	Codon optimized *alsS* and *alsD* from *B. subtilis* and *bdhA* from *S. cerevisiae* were integrated into *Pichia pastoris* genome and expressed under the control of GAP promoter.	(2R,3R)-2,3-BD	> 99	(21)
*E. coli*	A pathway for synthetic optically pure (2R,3R)-2,3-BD was constructed in *E. coli*	(2R,3R)-2,3-BD	99	(22)
*E. coli*	Expression of *alsS*, *alsD* and *budC* from *K. pneumoniae* in *E. coli*	meso-2,3-BD	100	(23)
*S. cerevisiae*	Deletion of *bdhA* and insert *budC* from *K. oxytoca* in *S. cerevisiae*	meso-2,3-BD	/	(24)
*E. coli* JM109	The genes including *ldhA*, *pta*, *adhE* and *poxB* were deleted from *E. coli* JM109 and cultured under hypoxic conditions.	meso-2,3-BD	/	(25)
*E. coli* YYC202	Overexpression of *ilvC*, *aldB* and *bdhA*	meso-2,3-BD	/	(26)
*E. coli*	Expression genes of *budB* and *budC* from *Enterobacter cloacae*	(2S,3S)-2,3-BD	95	(27)
*E. coli*	Overexpression of *gdh* and *fdh* in *E. coli*	(2S,3S)-2,3-BD	/	(28)
*L. lactis*	Expression gene of *budC* from *E. cloacae* in *L. lactis*	(2S,3S)-2,3-BD	100	(29)

Since *E. coli* lacks an endogenous AC and BD synthesis pathway, it is the most popular host strain from which optically pure AC and BD synthesis pathways are engineered. Xu et al. constructed a 3R-AC synthetic pathway in *E. coli* DH5 using acetoin biosynthetic genes (*budRAB*) from *S. marcescens* H30 and NADH oxidase genes (*nox*) from *Lactobacillus brevis* and the purity of 3R-AC stereoisomers reached 97.3% [68]. Ui et al. cloned three genes (*budB*, *budA*, and *budC*) of the BD synthesis operon in *K. pneumoniae* into *E. coli*, and recombinant *E. coli* was able to produce pure meso-2,3-BD from glucose [69]. Ji et al. cloned the *budB* and *budA* genes of *K. pneumoniae* and the *bdhA* gene of *B. subtilis* (encoding R-BDH) in *E. coli*, and the recombinant strain produced (2R,3R)-2,3-BD at 115 g/L and 99% optical purity [70].

Because (2S,3S)-2,3-BD cannot be synthesized directly from 3R-AC, it is uncommon in wild strains. DA is one of the precursors of 3S-AC and (2S,3S)-2,3-BD, and according to a previous analysis, it is derived from the nonenzymatic oxidation of α-acetolactate. Chu and his colleagues coexpressed ALS and S-BDH genes derived from *Enterobacter cloacae* in *E. coli*, thereby increasing the flux of α-acetyllactate to DA, which is first converted to 3S-AC and then to (2S,3S)-2,3-BD by S-BDH [71]. In addition, they introduced two NADH regenerating enzymes into the recombinant strain to further increase the concentration of DA and NADH and increase the yield of (2S,3S)-2,3-BD to 31.7 g/L [72]. This is the first engineered strain to produce (2S,3S)-2,3-BD using glucose as a carbon source.

Furthermore, *C. glutamicum*, *S. cerevisiae*, and *Pichia* are essential host organisms for synthetic biology. Mao and coworkers utilized *C. glutamicum* as the host, deleted its BDH gene and four byproduct synthesis genes (*pta*, *ack*, *ldh*, and *nagD*), and introduced *alsS* and *alsD* from *B. subtilis* 168. The recombinant strain produced 3R-AC with > 90% purity and a yield of 96.2 g/L [73]. As already mentioned, *S. cerevisiae* has two dehydrogenases involved in BD synthesis, but its BD production is low under normal physiological conditions. Lee et al. deleted its R-BDH gene and introduced the *budC* gene from *K. oxytoca*; the recombinant strain was able to produce 171 g/L of optically pure meso-2,3-BD [74]. Lian et al. constructed a novel BD biosynthetic pathway in *S. cerevisiae* by overexpressing its cytoplasmic α-acetolactate synthase gene (cytoILV2), *alsD* from *B. subtilis*, and its own R-BDH and the recombinant strain was able to produce 100 g/L of (2R,3R)-2,3-BD that was optically pure at 100% [75]. Yang et al. integrated the codon-optimized *alsS* and *alsD* genes from *B. subtilis* and the R-BDH gene from *S. cerevisiae* into the genome of *Pichia* pastoris and constructed a recombinant strain producing (2R, 3R)-2,3-BD with a purity greater than 99.9% [76].

### Resting cells and complex enzyme transformations

The production of optically pure isomers AC and BD catalyzed by resting cells or purified enzymes in vitro has also been extensively studied (Table 5). For instance, Xiao et al. expressed R-BDH from *B. subtilis* 168 and *Lactobacillus brevis* NADH oxidase (NOX, responsible for NAD^+^ regeneration) in *E. coli* BL21 (DE3) and prepared resting cells to synthesize 41.8 g/L 3R-AC with (2R,3R)-2,3-BD as the substrate and the purity reached 96% [77]. Guo et al. coexpressed genes of meso-BDH, NOX, and hemoglobin protein (VHB) from *Serratia* sp. T241 in *E. coli* and then used meso-2,3-BD as a substrate for transformation to obtain 3R-AC with 97.89% purity [78]. Yamada et al. expressed the gdh gene encoding glycerol dehydrogenase (GDH) from *Hansenula polymorpha* Dl-1 in *E. coli* HB101 and prepared resting cells to catalyze (2R,3R)-2,3-BD to obtain 3R-AC with > 99.9% purity [79]. This is in keeping with the earlier statement that GDH has R-BDH activity. Cui used heterologously expressed meso-BDH and xylose reductase from Candida tenuis to form a dual enzyme system to catalyze meso-2,3-BD and xylose production of 3R-AC [80].

**Table 5:** Optically pure AC and BD were obtained by resting cells and complex enzyme transformations

Strains	Methods	Substrate	Product	Enantiomeric excess (%)	Reference
*E. coli* HB101	Expression of *gdh* from *Hansenula polymorpha* Dl-1	(2R,3R)-2,3-BD	3R-AC	> 99.9	(30)
*E. coil* BL21(DE3)	Expression of *bdhA* from *B. subtilis* 168 and *nox* from *L. brevis* CICC 6004	(2R,3R)-2,3-BD	3R-AC	96	(31)
*E. coli*	Expression of *budC* from *C. glutamicum* ATCC13032 and *xyl1* from *Candida tenuis*	meso-2,3-BD	3R-AC	/	(32)
*E. coli* BL21 (DE3)	Co-expression of *budC*, *nox* and *vhb* from *Serratia* sp. T241	meso-2,3-BD	3R-AC	97.89	(33)
*E. coli* Rosetta	Overexpression of *dar* from *P. polymyxa ZJ-9*	DA	3S-AC	> 99.9	(34)
*E. coli* BL21(DE3)	Expression of *dar* with high stereoselectivity, specificity and stability and the in situ-NADH regeneration system	DA	3S-AC	99.5	(35)
*K. pneumoniae* CICC 10,011 and *B. subtilis* 168	* K. pneumoniae* CICC 10,011 resting cells convert glucose to a mixture of meso-2,3-BD and (2 S,3 S)-2,3-BD. *B. subtilis* 168 resting cells convert meso-2,3-BD in the mixture to 3 S-AC	Glucose	3S-AC	96.2	(36)
*E. coli* BL21(DE3)	Expressing *bdhA* from *B. subtilis* 168 and *nox* from *L. brevis* CICC 6004	(2R,3R)-2,3-BD	3R-AC	96	(31)
		meso-2,3-BD	(2S,3S)-2,3-BD	99	
*E. coli*	Expressing *dar* from *Enterobacter cloacae* sp.	DA	(2S,3S)-2,3-BD	> 99	(13)

As mentioned previously, direct production of 3S-AC by microbial fermentation is challenging, and the biocatalytic route is the most common method for producing 3S-AC. Diacetyl is a direct precursor of 3S-AC and can be converted to 3S-AC by being catalyzed by DAR. For instance, Gao et al. successfully obtained 3S-AC with > 99.9% optical purity by transforming DA with recombinant *E. coli* resting cells and expressing the DAR gene of the *P. polymyxa* ZJ-9 strain in *E. coli* [81]. DA can also be utilized to synthesize (2S,3S)-2,3-BD. For example, Li et al. cloned the BDH gene from *Enterobacter cloacae* sp. in *E. coli*, and recombinant *E. coli* whole cells can synthesize (2S,3S)-2,3-BD using DA. Under optimal reaction conditions, (2S,3S)-2,3-BD of greater than 99% optical purity was produced [82]. Compared to 3S-AC, meso-2,3-BD is easier to acquire. Liu et al. first converted glucose into a mixture of meso-2,3-BD and (2S,3S)-2,3-BD using resting cells of *K. pneumoniae* CICC 10,011, and then used resting cells of *B. subtilis* 168 to convert meso-2,3-BD into 3S-AC with a purity of 96.2%, while obtaining a purity of 99% for (2S,3S)-2,3-BD [83].

Current research on in vitro catalysis primarily employs resting cells to produce 3R-AC or 3S-AC, and the substrates are (2R, 3R)-2, 3-BD, meso-BD, and DA, all of which require high preparation costs; therefore, the preparation of optically pure AC and BD by resting cells or complex enzymes is not economically viable for industrial production.

## Conclusion

In chemical synthesis and drug synthesis, the optical isomers AC and BD have important industrial applications. Many microorganisms can accumulate large amounts of AC or BD in fermentation broth using glucose as the raw material, but usually these AC or BD are a mixture of various optical isomers, which was found to be due to the simultaneous presence of more than one dehydrogenase with BDH properties in microorganisms. This paper begins with the synthesis pathway of bacterial AC and BD, reviews the formation mechanism of different stereoisomers of AC and BD in detail, and summarizes the properties of different types of BDH that have been demonstrated. The structural characteristics and genetic relationships of different types of BDH were compared and analyzed. Simultaneously, the works done in recent years to produce optically pure AC or BD using microorganisms were summarized. According to the summary and analysis presented above, there are four major dehydrogenases involved in the synthesis of AC or BD optically pure isomers, which can be divided into two major groups: R-BDH is the only member of MDR, while meso-BDH, S-BDH, and DAR are all members of SDR. With the participation of the abovementioned dehydrogenases and NAD(H), interconversion between the various stereoisomeric optically pure isomers of AC and BD is possible (Fig. 3); therefore, high optically pure AC and BD production strains can be obtained by pathway modification and reconstitution. As far as the fermentation results of the reported engineered strains are concerned, the optical purity of some AC and BD strains can reach more than 99%, whereas the optical purity of the majority of AC and BD strains is approximately 95% or even as low as 90%. The low specificity of the chosen BDH substrate and the nonenzymatic oxidation of α-acetolactate in the host strains may have contributed to this result. Furthermore, there may be additional unidentified oxidoreductases involved in the AC and BD conversion of the various stereoisomers in the recombinant strain. Therefore, to use microorganisms to produce high-optical-purity AC and BD products efficiently and directionally, the breeding and construction of single enzyme chassis strains and the analysis of the mechanism of BDH in catalytic reactions are essential.

### Electronic supplementary material

Below is the link to the electronic supplementary material.

Supplementary Material 1

## Data Availability

The data that support the findings of this study are available from the corresponding author, [Zhao, X], upon reasonable request.
