# Supplementary material for: Mechanism of microbial production of acetoin and 2,3-butanediol optical isomers and substrate specificity of butanediol dehydrogenase
Source: Microb Cell Fact. 2023 Aug 29;22:165. doi: 10.1186/s12934-023-02163-6 (PMC10466699; doi:10.1186/s12934-023-02163-6)
Supplement: Supplementary file 1 — Supplementary Material 1 [file 12934_2023_2163_MOESM1_ESM.docx]

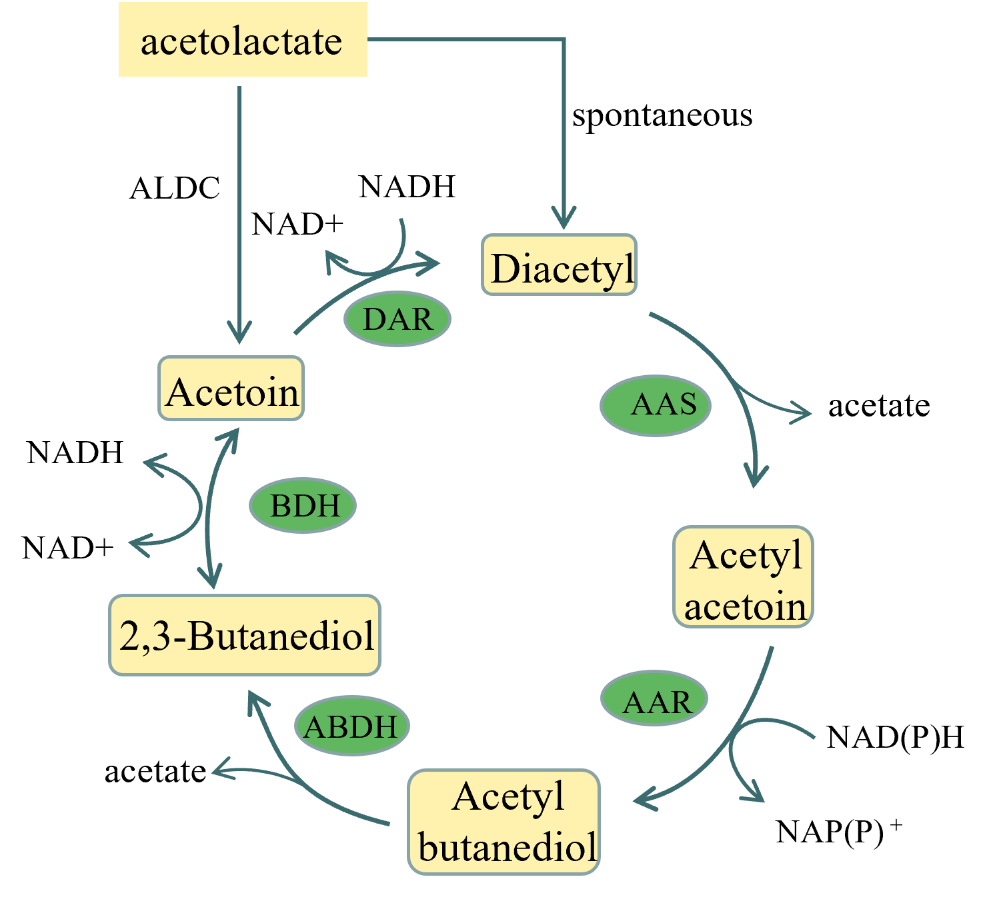


**Supplementary Figure A**. 2,3-butanediol cycle. AAS, Acetylacetoin synthase; AAR, Acetylacetoin reductase; ABDH, Acetylbutanediol hydrolase; BDH, 2,3-butanediol dehydrogenase; DAR, Diacetyl reductase.

**
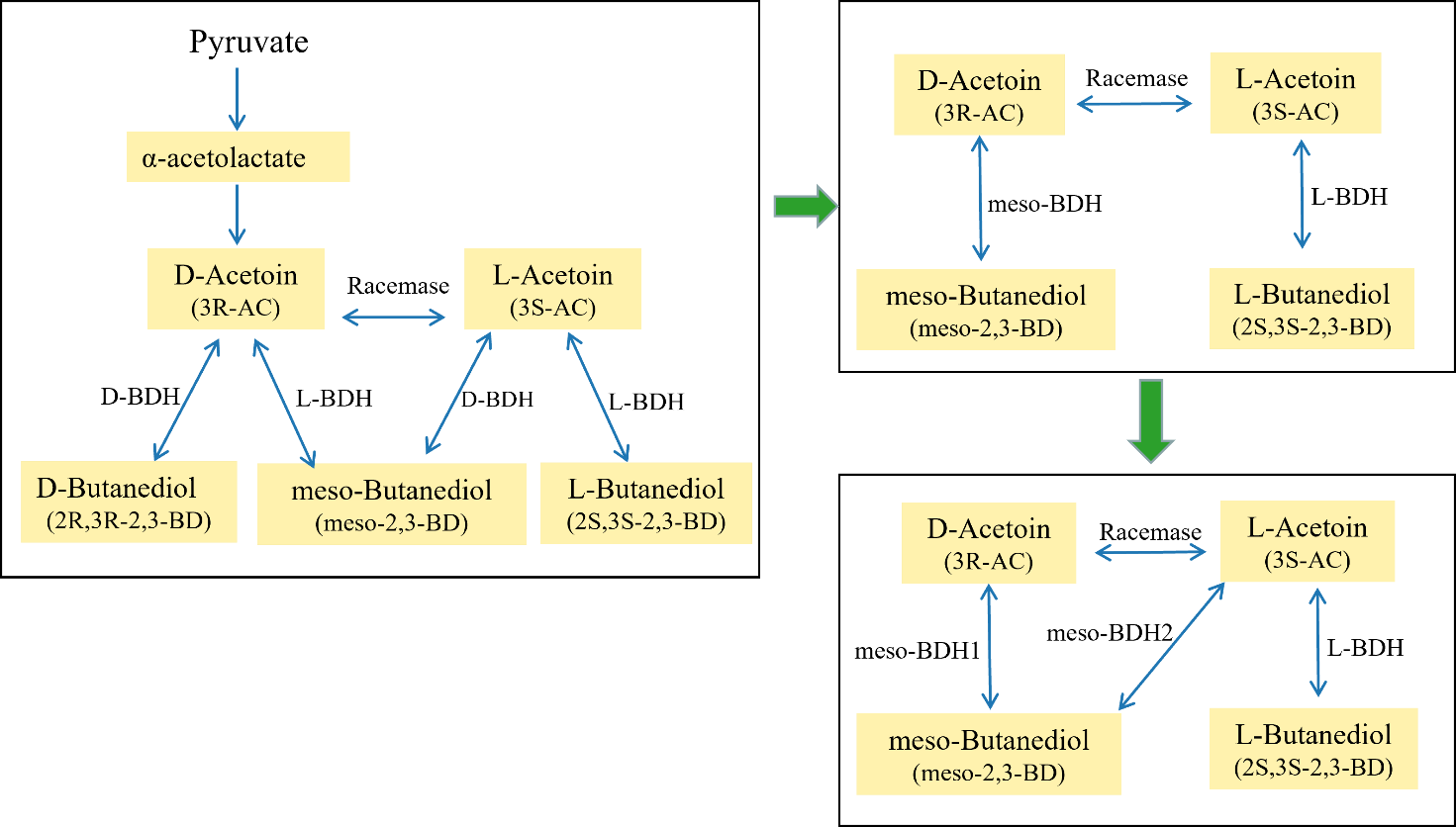
**

**Supplementary Figure B.** Early formation mechanism of different optical isomers of AC and BD.

**Supplementary Table A.1** Comparison of R-BDH sequences. Alignment of the sequence from the R-BDH of *Bacillus subtilis* 168.

| **Organism** | **Enzyme** | **Length (aa)** | **Amino acid identity (%)** | **Amino acid similarity (%)** |
| --- | --- | --- | --- | --- |
| *Bacillus subtilis* 168 | R-BDH | 346 | 100 | 100 |
| *Paenibacillus polymyxa* | R-BDH | 350 | 69.2 | 83.8 |
| *Rhodococcus erythropolis* | R-BDH | 351 | 49 | 62.8 |
| *Brevibacterium aurantiacum* | R-BDH | 353 | 43.9 | 59.5 |
| *Glutamicibacter arilaitensis DSM 16368* | R-BDH | 353 | 44.5 | 59.4 |
| *Mycobacterium* sp. B-009 | R-BDH | 348 | 42.1 | 59 |
| *Saccharomyces cerevisiae* S288c | R-BDH | 382 | 35.1 | 52.1 |

**Supplementary Table A.2** Sequence comparison among meso-BDH, S-BDH and DAR. Alignment of the sequence from the meso-BDH of *Klebsiella pneumoniae.*

| **Organism** | **Enzyme** | **Length (aa)** | **Amino acid identity (%)** | **Amino acid similarity (%)** |
| --- | --- | --- | --- | --- |
| *Klebsiella pneumoniae* | meso-BDH | 256 | 100 | 100 |
| *Enterobacter ludwigii* | meso-BDH | 264 | 86.4 | 90.9 |
| *Bacillus licheniformis* | meso-BDH | 260 | 66.2 | 77.7 |
| *Bacillus* sp. | meso-BDH | 248 | 33.1 | 48.3 |
| *Mycobacterium dioxanotrophicus* | meso-BDH | 252 | 30.9 | 46 |
| *Salipiger profundus* | meso-BDH | 252 | 29.1 | 47 |
| *Corynebacterium glutamicum* | S-BDH | 258 | 48.8 | 67.8 |
| *Leuconostoc inhae* | S-BDH | 258 | 45.7 | 57.8 |
| *Azospirillum* sp. OGB3 | S-BDH | 261 | 45.5 | 54.9 |
| *Rhizobium soli* | S-BDH | 253 | 34.5 | 49.1 |
| *Microbacterium* sp. SA39 | S-BDH | 270 | 29.3 | 44.9 |
| *Enterobacter* sp. MGH 24 | DAR | 256 | 89.8 | 93.4 |
| *Acinetobacter* sp. WC-743 | DAR | 256 | 70.4 | 80.2 |
| *Lactococcus lactis* | DAR | 256 | 50 | 65.2 |
| *Leuconostoc carnosum* JB16 | DAR | 253 | 50 | 64.8 |

**Supplementary Table A.3** Sequence comparison among S-BDH and DAR. Alignment of the sequence from the S-BDH of Corynebacterium glutamicum*.*

| **Organism** | **Enzyme** | **Length (aa)** | **Amino acid identity (%)** | **Amino acid similarity (%)** |
| --- | --- | --- | --- | --- |
| *Corynebacterium glutamicum* | S-BDH | 258 | 100 | 100 |
| *Leuconostoc carnosum* JB16 | DAR | 253 | 51 | 68 |
| *Enterobacter* sp. MGH 24 | DAR | 256 | 48.9 | 67.0 |
| *Lactococcus lactis* | DAR | 256 | 45.2 | 66.8 |
| *Acinetobacter* sp. WC-743 | DAR | 256 | 44.5 | 65 |

1. Elmahmoudy M, Elfeky N, Zhongji P, Zhang Y, Bao Y. 2021. Identification and characterization of a novel 2R,3R-Butanediol dehydrogenase from Bacillus sp. DL01. Electronic Journal of Biotechnology 49:56-63.

2. Yu M, Huang M, Song Q, Shao J, Ying X. 2015. Characterization of a (2R,3R)-2,3-Butanediol Dehydrogenase from Rhodococcus erythropolis WZ010. Molecules 20:7156-73.

3. Gao J, Yang HH, Feng XH, Li S, Xu H. 2013. A 2,3-butanediol dehydrogenase from Paenibacillus polymyxa ZJ-9 for mainly producing R,R-2,3-butanediol: purification, characterization and cloning. J Basic Microbiol 53:733-41.

4. Gong F-Q, Liu Q-S, Tan H-D, Li T, Tan C-Y, Yin H. 2019. Cloning, expression and characterization of a novel (2R,3R) -2,3-butanediol dehydrogenase from Bacillus thuringiensis. Biocatalysis and Agricultural Biotechnology 22.

5. Zhang GL, Wang CW, Li C. 2012. Cloning, expression and characterization of meso-2,3-butanediol dehydrogenase from Klebsiella pneumoniae. Biotechnol Lett 34:1519-23.

6. Zhang L, Xu Q, Zhan S, Li Y, Lin H, Sun S, Sha L, Hu K, Guan X, Shen Y. 2014. A new NAD(H)-dependent meso-2,3-butanediol dehydrogenase from an industrially potential strain Serratia marcescens H30. Appl Microbiol Biotechnol 98:1175-84.

7. Xu GC, Bian YQ, Han RZ, Dong JJ, Ni Y. 2016. Cloning, Expression, and Characterization of budC Gene Encoding meso-2,3-Butanediol Dehydrogenase from Bacillus licheniformis. Appl Biochem Biotechnol 178:604-17.

8. Lv X, Dai L, Bai F, Wang Z, Zhang L, Shen Y. 2016. Metabolic engineering of Serratia marcescens MG1 for enhanced production of (3R)-acetoin. Bioresour Bioprocess 3:52.

9. Wang D, Zhou J, Chen C, Wei D, Shi J, Jiang B, Liu P, Hao J. 2015. R-acetoin accumulation and dissimilation in Klebsiella pneumoniae. J Ind Microbiol Biotechnol 42:1105-15.

10. Lu L, Mao Y, Kou M, Cui Z, Jin B, Chang Z, Wang Z, Ma H, Chen T. 2020. Engineering central pathways for industrial-level (3R)-acetoin biosynthesis in Corynebacterium glutamicum. Microb Cell Fact 19:102.

11. Guo Z, Zhao X, He Y, Yang T, Gao H, Li G, Chen F, Sun M, Lee JK, Zhang L. 2016. Efficient (3R)-acetoin production from meso-2,3-butanediol using a new whole-cell biocatalyst with co-expression of meso-2,3-butanediol dehydrogenase, NADH oxidase and Vitreoscilla hemoglobin. Journal of Microbiology & Biotechnology.

12. Bai F, Dai L, Fan J, Truong N, Rao B, Zhang L, Shen Y. 2015. Engineered Serratia marcescens for efficient (3R)-acetoin and (2R,3R)-2,3-butanediol production. J Ind Microbiol Biotechnol 42:779-86.

13. Li L, Wang Y, Zhang L, Ma C, Wang A, Tao F, Xu P. 2012. Biocatalytic production of (2S,3S)-2,3-butanediol from diacetyl using whole cells of engineered Escherichia coli. Bioresour Technol 115:111-6.

14. Zhang L, Cao C, Jiang R, Xu H, Xue F, Huang W, Ni H, Gao J. 2018. Production of R,R-2,3-butanediol of ultra-high optical purity from Paenibacillus polymyxa ZJ-9 using homologous recombination. Bioresour Technol 261:272-278.

15. Park JM, Rathnasingh C, Song H. 2015. Enhanced production of (R,R)-2,3-butanediol by metabolically engineered Klebsiella oxytoca. J Ind Microbiol Biotechnol 42:1419-25.

16. Lian J, Chao R, Zhao H. 2014. Metabolic engineering of a Saccharomyces cerevisiae strain capable of simultaneously utilizing glucose and galactose to produce enantiopure (2R,3R)-butanediol. Metab Eng 23:92-9.

17. Ge Y, Li K, Li L, Gao C, Zhang L, Ma C, Xu P. 2016. Contracted but effective: production of enantiopure 2,3-butanediol by thermophilic and GRAS Bacillus licheniformis. Green Chemistry 18:4693-4703.

18. Fu J, Huo G, Feng L, Mao Y, Wang Z, Ma H, Chen T, Zhao X. 2016. Metabolic engineering of Bacillus subtilis for chiral pure meso-2,3-butanediol production. Biotechnol Biofuels 9:90.

19. Xu Q, Xie L, Li Y, Lin H, Sun S, Guan X, Hu K, Shen Y, Zhang L. 2015. Metabolic engineering ofEscherichia colifor efficient production of (3R)-acetoin. Journal of Chemical Technology & Biotechnology 90:93-100.

20. Mao Y, Fu J, Tao R, Huang C, Wang Z, Tang Y-J, Chen T, Zhao X. 2017. Systematic metabolic engineering of Corynebacterium glutamicum for the industrial-level production of optically pure d-(−)-acetoin. Green Chemistry 19:5691-5702.

21. Yang Z, Zhang Z. 2018. Production of (2R, 3R)-2,3-butanediol using engineered Pichia pastoris: strain construction, characterization and fermentation. Biotechnol Biofuels 11:35.

22. Tong YJ, Ji XJ, Shen MQ, Liu LG, Nie ZK, Huang H. 2016. Constructing a synthetic constitutive metabolic pathway in Escherichia coli for (R, R)-2,3-butanediol production. Appl Microbiol Biotechnol 100:637-47.

23. Sadaharu, Ui, and, Yoshifumi, Okajima, and, Akio, Mimura, and, Haruhiko. 1997. Molecular generation of an Escherichia coli strain producing only the meso-isomer of 2,3-butanediol - ScienceDirect. Journal of Fermentation and Bioengineering 84:185-189.

24. Lee YG, Bae JM, Kim SJ. 2022. Enantiopure meso-2,3-butanediol production by metabolically engineered Saccharomyces cerevisiae expressing 2,3-butanediol dehydrogenase from Klebsiella oxytoca. J Biotechnol 354:1-9.

25. Li ZJ, Jian J, Wei XX, Shen XW, Chen GQ. 2010. Microbial production of meso-2,3-butanediol by metabolically engineered Escherichia coli under low oxygen condition. Appl Microbiol Biotechnol 87:2001-9.

26. Nielsen DR, Yoon SH, Yuan CJ, Prather KL. 2010. Metabolic engineering of acetoin and meso-2, 3-butanediol biosynthesis in E. coli. Biotechnol J 5:274-84.

27. Chu H, Xin B, Liu P, Wang Y, Li L, Liu X, Zhang X, Ma C, Xu P, Gao C. 2015. Metabolic engineering of Escherichia coli for production of (2S,3S)-butane-2,3-diol from glucose. Biotechnol Biofuels 8:143.

28. Wang Y, Li L, Ma C, Gao C, Tao F, Xu P. 2013. Engineering of cofactor regeneration enhances (2S,3S)-2,3-butanediol production from diacetyl. Sci Rep 3:2643.

29. Liu J, Chan SHJ, Brock-Nannestad T, Chen J, Lee SY, Solem C, Jensen PR. 2016. Combining metabolic engineering and biocompatible chemistry for high-yield production of homo-diacetyl and homo-(S,S)-2,3-butanediol. Metab Eng 36:57-67.

30. Yamada-Onodera K, Yamamoto H, Kawahara N, Tani Y. 2010. Expression of the Gene of Glycerol Dehydrogenase from Hansenula polymorpha Dl-1 in Escherichia coli for the Production of Chiral Compounds. Engineering in Life Sciences 22:355-362.

31. Xiao Z, Lv C, Gao C, Qin J, Ma C, Liu Z, Liu P, Li L, Xu P. 2010. A novel whole-cell biocatalyst with NAD+ regeneration for production of chiral chemicals. PLoS One 5:e8860.

32. Cui Z, YufengZhao, YujiaoChen, CongTang, Ya-JieChen, TaoMa, HongwuWang, Zhiwen. 2018. Concomitant cell-free biosynthesis of optically pure D-(-)-acetoin and xylitol via a novel NAD(+) regeneration in two-enzyme cascade. Journal of Chemical Technology & Biotechnology 93.

33. Guo Z, Zhao X, He Y, Yang T, Gao H, Li G, Chen F, Sun M, Lee JK, Zhang L. 2017. Efficient (3R)-Acetoin Production from meso-2,3-Butanediol Using a New Whole-Cell Biocatalyst with Co-Expression of meso-2,3-Butanediol Dehydrogenase, NADH Oxidase, and Vitreoscilla Hemoglobin. J Microbiol Biotechnol 27:92-100.

34. Gao J, Xu YY, Li FW, Ding G. 2013. Production of S-acetoin from diacetyl by Escherichia coli transformant cells that express the diacetyl reductase gene of Paenibacillus polymyxa ZJ-9. Lett Appl Microbiol 57:274-81.

35. Li JX, Huang YY, Chen XR, Du QS, Meng JZ, Xie NZ, Huang RB. 2018. Enhanced production of optical (S)-acetoin by a recombinant Escherichia coli whole-cell biocatalyst with NADH regeneration. RSC Adv 8:30512-30519.

36. Liu Z, Qin J, Gao C, Hua D, Ma C, Li L, Wang Y, Xu P. 2011. Production of (2S,3S)-2,3-butanediol and (3S)-acetoin from glucose using resting cells of Klebsiella pneumonia and Bacillus subtilis. Bioresour Technol 102:10741-4.
